# The Stimulus Selectivity and Connectivity of Layer Six Principal Cells Reveals Cortical Microcircuits Underlying Visual Processing

**DOI:** 10.1016/j.neuron.2014.09.026

**Published:** 2014-10-01

**Authors:** Mateo Vélez-Fort, Charly V. Rousseau, Christian J. Niedworok, Ian R. Wickersham, Ede A. Rancz, Alexander P.Y. Brown, Molly Strom, Troy W. Margrie

(Neuron *83*, 1431–1443; September 17, 2014)

As the result of a production error, the final sentence of the Intrinsic Parameters section of the Experimental Procedures erroneously referred to “normalized and nonnormalized data” instead of “normally and nonnormally distributed data.” This sentence has been corrected both in the print issue and online, and the journal regrets the error.

