# Supplementary material for: The Stimulus Selectivity and Connectivity of Layer Six Principal Cells Reveals Cortical Microcircuits Underlying Visual Processing
Source: Neuron. 2014 Sep 17;83(6):1431–43. doi: 10.1016/j.neuron.2014.08.001 (PMC4175007; doi:10.1016/j.neuron.2014.08.001)
Supplement: Document S1. Supplemental Experimental Procedures and Figures S1–S3 [file mmc1.pdf]

Neuron, Volume *83*

Supplemental Information

The Stimulus Selectivity and Connectivity of Layer Six Principal Cells Reveals Cortical Microcircuits Underlying Visual Processing

Mateo Vélez-Fort, Charly V. Rousseau, Christian J. Niedworok, Ian R. Wickersham, Ede A. Rancz, Alexander P.Y. Brown, Molly Strom, and Troy W. Margrie

Supplemental Figures, Movies, Experimental Procedures and References

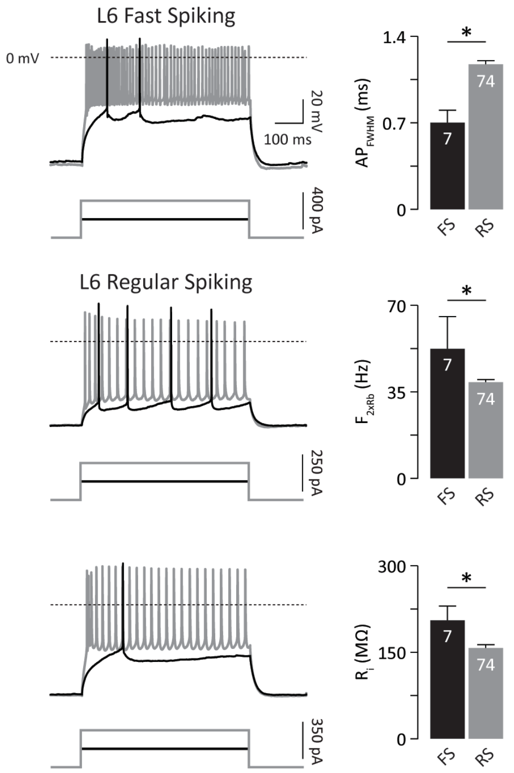

Supplementary Figure 1

Figure S1 (related to Figure 1): Intrinsic properties of recorded fast and regular spiking L6 cells Left column: Example traces recorded from fast (top) and regular spiking (middle and bottom) neurons recorded at the rheobase (black) and 2x the rheobase (grey). Right: Bar graphs comparing the AP width at half the peak amplitude (AP_{FWHM}), average frequency of firing at 2x the rheobase (F_{2xRb}) and input resistance (R_i).

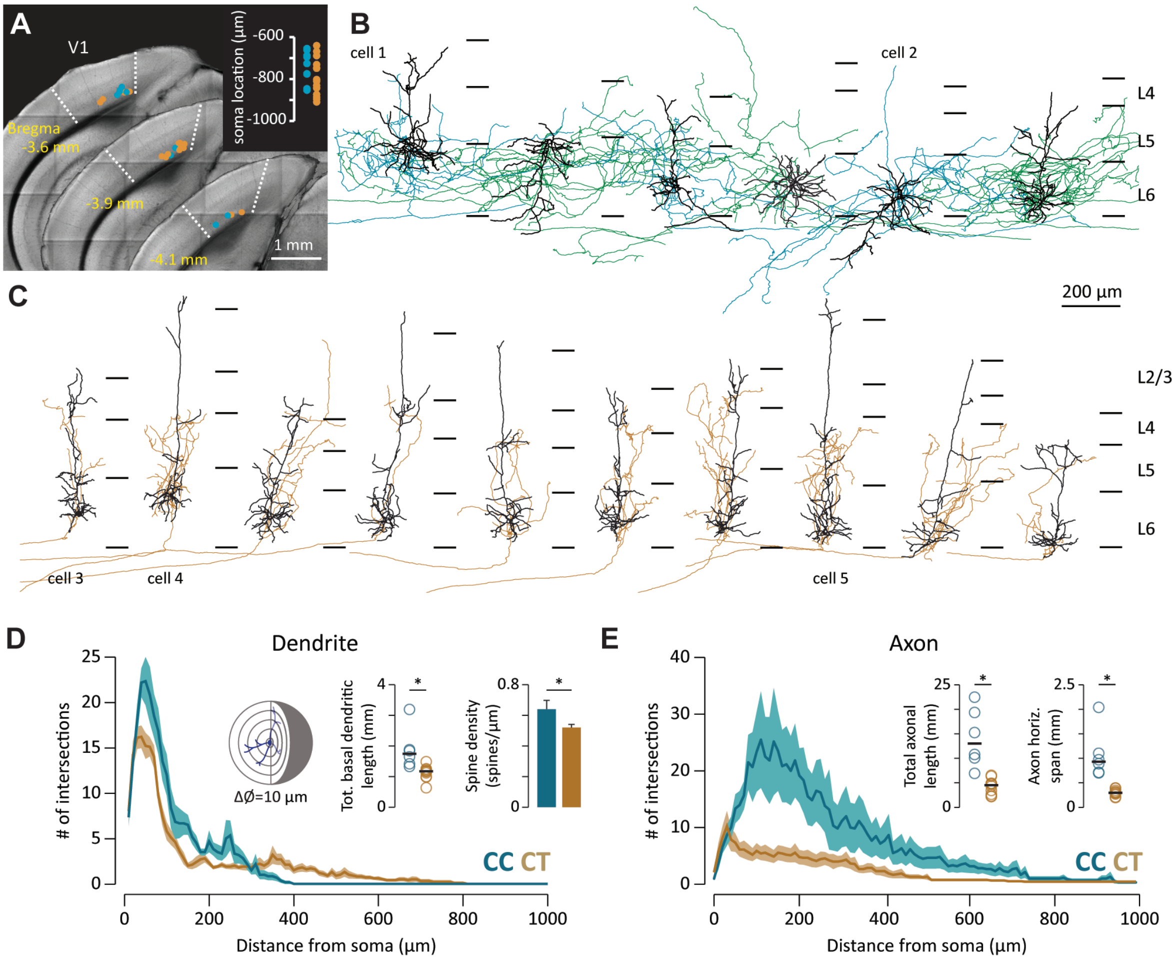

Supplementary Figure 2

Figure S2 (related to Figure 1): Morphological classification of *in vivo*-recorded L6 principal cells

(**A**) Three consecutive "reference brain" slices showing the location of the reconstructed cells in B and C recorded from individual brains. Inset indicates the shortest distance of the cell soma from the pia. (**B**) Reconstructions of recorded neurons with dendrites that do not project beyond layer 4 and have elaborate axonal branching within the cortex. (**C**) Morphologies of recorded cells that extended their dendrites beyond layer 4 and always projected their axons into the thalamic tract. (**D**) Sholl analysis showing the number of concentric sphere crossings made by CC (blue) and CT (brown) dendritic arborizations plotted against their distance from the cell soma. Insets show the total basal dendritic for each reconstructed CC and CT cell (left), the horizontal bar indicates the median value. Right, histogram of the mean spine density for CC (n = 3, 12 segments) and CT cells (n = 4, 20 segments). (**E**) Sholl analysis showing the number of concentric sphere crossings made by CC (blue) and CT (brown) axonal arborizations plotted against their distance from the cell soma. Insets show the total axonal length (left) and medial-lateral (horizontal) span for CC versus CT axons. The median is indicated by the horizontal bar.

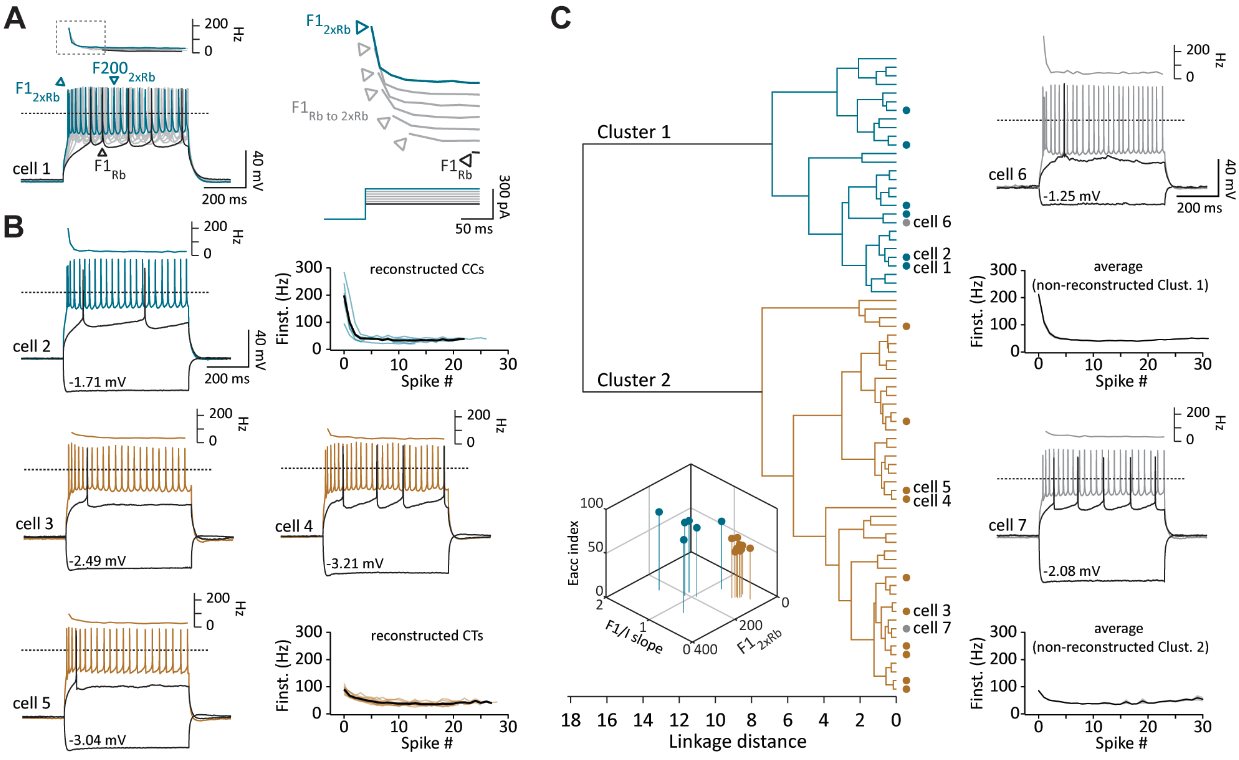

Supplementary Figure 3

Figure S3 (related to Figure 1): Biophysical classification of recorded cells verified by morphological identity

(**A**) Example of membrane voltage traces from a reconstructed CC cell showing the biophysical parameters used to perform the cluster analysis in (C). $F1_{Rb}$ = instantaneous frequency of the first two spikes evoked by current step injection at the rheobase. $F1_{2xRb}$ = instantaneous frequency of the first two spikes evoked at 2x the rheobase. $F200_{2xRb}$ = the instantaneous firing frequency 200 ms after the onset of the current injection at 2x the rheobase. Top: Plot of the instantaneous firing frequency evoked by current steps ranging from the rheobase (black) to 2x the rheobase (blue). Right: Example traces highlighted by the dashed box showing the relationship between F1 and the current step amplitude. (**B**) Example membrane voltage traces from one CC (blue) and three CT (brown) reconstructed cells showing spiking at the rheobase and 2x the rheobase. Above: Plot of the instantaneous firing frequency evoked by current step injection at 2x the rheobase. Insets: The average instantaneous firing rates recorded at 2x the rheobase plotted against spike number for all reconstructed CC (blue) and CT cells (brown). (**C**) Left: Result of the cluster analysis using the membrane sag and the three firing parameters indicated in the 3D plot (insert). Clustered cells with known morphology are indicated by filled blue (CC) and brown (CT) circles. Right: The average instantaneous firing rates recorded at 2x the rheobase plotted against spike number for all cells in the respective cluster (except the reconstructed cells). Example traces from individual non-reconstructed cells selected from cluster 1 (top) and cluster 2 (below) showing spiking at rheobase and 2x the rheobase.

Movie S1 (related to Figure 1): 3D projection showing the dendritic density for CC and CT cells. Movies are the sum of dendritic reconstructions of six and ten in vivo-recorded cells (CC and CT respectively) centered on the neuronal somata and vertically aligned according to the shortest distance to the pia surface. The dendritic density is expressed in false color (see Figure 1B).

Movie S2 (related to Figure 1): 3D projection showing the axonal density for CC and CT cells. Movies are the sum of the axonal reconstructions of six and ten in vivo-recorded cells (CC and CT respectively) centered on the neuronal somata and vertically aligned according to the shortest distance to the pia surface. The axonal density is expressed in false color (see Figure 1B).

Movie S3 (related to Figures 5 and 6): Raw images of retrograde labeled RFP-expressing cells. Top: Stacks of 50 coronal images (every 5 μm (Z)) showing modified rabies-infected neurons within the deep layers of V1 after a single-CC cell rabies tracing experiment (left: native RFP, right: spheres localized using the coordinates of manually counted RFP-expressing cells). Bottom: Stacks showing cells within the deep layers of V1 after a single-CT cell rabies tracing experiment (left: native RFP, right: spheres localized using the coordinates of manually counted RFP-expressing cells).

Experimental Procedures

Tissue processing

Deeply anaesthetised mice were transcardially perfused with cold PB (0.1 M) followed by 4% paraformaldehyde (PFA) in PB (0.1 M) and the brain left overnight in 4% PFA at 4 °C. Coronal brain sections (50 - 150 µm) were either cut directly on a vibrating microtome (Leica VT1200S or Microm HM 650V) or obtained following serial tomography, then rinsed in PBS. Fluorescence images were acquired on a Leica SP1, SP5 or a Zeiss 510 laser-scanning confocal microscope. For experiments containing biocytin in the intracellular solution, slices were either treated for streptavidin or 3,3-diaminobenzidine (DAB) labeling. For streptavidin labeling, slices were first treated with 5% bovine serum albumin and 2% Triton-X100 in PBS for 12 hours then incubated in a PBS solution containing 0.5% Alexa-488-conjugated streptavidin (Sigma-Aldrich), 5% bovine serum albumin and 0.1% Triton-X for 12 hours at 4˚C. For the DAB procedure, slices were incubated in H_2O_2 (3% wt/vol) for 30 minutes then rinsed with PB and incubated in a solution containing 0.1% triton-X100 and avidin-biotinylated horseradish peroxidase (Vector ABC staining kit, Vector Lab) for 12 hours at 4˚C. Finally, slices were rinsed with PB and incubated for 30 minutes in DAB solution; H_2O_2 (3% wt/vol) was added under visual control until neuronal cell processes were visible then slices were rinsed with PB and mounted (see below). For immunostaining against GFP and RFP, chicken anti-GFP (Life Technologies 1:500) and rabbit anti-tRFP primary antibodies (Evrogen 1:1000; 12 hours at RT) were visualized with goat DyLight-488 anti-chicken (Abcam 1:500) and goat Alexa Fluor-555 anti-rabbit (Life Technologies 1:500, 12 hours at RT) secondary antibodies, respectively. After thorough washing in PB, slices were mounted with an anti-fading medium (Mowiol 4-88, Calbiochem + 2.5% DABCO 33-LV, Sigma-Aldrich).

Assignment of cortical layers

Cortical layers in V1 were manually assigned using either DAPI staining or the background fluorescence (cell nuclei appear as shadows) to qualitatively use cell densities to identify laminar boundaries. Boundary assignment was independently performed by four postgraduate and postdoctoral cortical neuroscientists in the laboratory. The maximum discrepancy in layer assignment was 52 µm which was at the layer 5/6 boundary. Across all layer boundaries the average maximum discrepancy was 26 µm.

Plasmids and viruses

Production

Plasmids pSil-ETN-IRES2-EGFP (protein expressed from ETN is a fusion of the extracellular and trans-membrane domains of the rabies glycoprotein from the CVS-N2c strain and the intracellular domain from the SADB19 glycoprotein, Addgene 58831) and pSil-TVA800-IRES2-EGFP were made by replacing GFP in pCA-b-GFPm5 silencer 3 (generous gift of Hendrik Wildner and François Guillemot) by the indicated cassettes to enable expression in neurons from CAGG promoter. Human XIAP cDNA was made by RT-PCR (Quantitect, Qiagen) amplification using oligonucleotides (5' ccacgtctctggccaagatgacttttaacagttttg and 5' ccacgtctcggatccgctaatggaattcaatcctg) and cloned into pCA-b-GFPm5 silencer 3 replacing GFP (Addgene, 58832). The plasmid pAAV-EF1a-Flex-GT is from Addgene (26198). The plasmid pAAV-EF1a-Flex-C-RG was made by replacing GT in pAAV-EF1a-Flex-GT with Cerulean-E2A-SADB19 rabies glycoprotein (RV-G) (Addgene, 49101). pAAV2/8 was obtained from Penn Vector Core and pHelper and AAV293 cells were purchased from Agilent Technologies. AAV virus was prepared from transfection of 5 x 100 mm dishes as per manufacturer's protocol (Agilent Technologies) and were purified from cell pellets as described (Guo et al., 2012) through to PEG precipitation. Virus containing pellets were re-suspended by trituration and overnight incubation at 4°C in 1xHBSS (Invitrogen), extracted with an equal volume of chloroform, and concentrated using

Amicon Ultra-Ultracel 100 K filters (Millipore) to a final volume of approximately 100 µl. EnvA pseudotyped SADB19 rabies virus expressing tagRFP in place of the RG was prepared from EnvA-RV-tagRFP by infecting cells expressing TVA950 and RG (expression plasmids were generous gifts from Melvyn Yap and Martin Schwarz, respectively) to amplify the unpseudotyped virus. This stock was used to re-pseudotype virus with EnvA according to published methods (Wickersham et al., 2007). All reagents for cell culture were from Invitrogen, BHK cells and BHK-EnvARGCD cells were a generous gift of Martin Schwartz.

Injections

For virus injection, long-shanked, volume-calibrated pipettes (Blaubrand) were pulled and tip-filled using negative pressure (Cetin et al., 2006). To ensure the injection site was close to the recorded cell, pipettes were mounted onto the recording manipulator on the same setup used for whole-cell recording and plasmid delivery. Approximately 50 nl of modified rabies virus solution was injected under 30-50 mbar of positive pressure for 3 minutes. The pipette was withdrawn, Kwik-Sil re-applied and the animal sutured and recovered. For cre-NTSR1 mice, a first injection of approximately 20 nl of the helper virus (see below) was followed 2 days later by a second injection of approximately 50 nl of the modified rabies virus solution. Post-RV injection mice were maintained for 10-12 days.

Supplemental References

Cetin, A., Komai, S., Eliava, M., Seeburg, P.H., and Osten, P. (2006). Stereotaxic gene delivery in the rodent brain. Nat. Protoc. *1*, 3166-3173.

Guo, P., El-Gohary, Y., Prasadan, K., Shiota, C., Xiao, X., Wiersch, J., Paredes, J., Tulachan, S., and Gittes, G.K. (2012). Rapid and simplified purification of recombinant adeno-associated virus. J. Virol. Methods *183*, 139-146.

Wickersham, I.R., Finke, S., Conzelmann, K.K., and Callaway, E.M. (2007). Retrograde neuronal tracing with a deletion-mutant rabies virus. Nat. Methods *4*, 47-49.
