# Supplementary material for: The Stimulus Selectivity and Connectivity of Layer Six Principal Cells Reveals Cortical Microcircuits Underlying Visual Processing
Source: Neuron. 2014 Sep 17;83(6):1431–43. doi: 10.1016/j.neuron.2014.08.001 (PMC4175007; doi:10.1016/j.neuron.2014.08.001)
Supplement: Document S2. Article plus Supplemental Information [file mmc5.pdf]

Neuron
Article

The Stimulus Selectivity and Connectivity of Layer Six Principal Cells Reveals Cortical Microcircuits Underlying Visual Processing

Mateo Vélez-Fort,[1] Charly V. Rousseau,[1] Christian J. Niedworok,[1] Ian R. Wickersham,[2] Ede A. Rancz,[1] Alexander P.Y. Brown,[1] Molly Strom,[1] and Troy W. Margrie[1,3,*]
[1]The Division of Neurophysiology, MRC National Institute for Medical Research, Mill Hill, London NW7 1AA, UK
[2]Genetic Neuroengineering Group, Massachusetts Institute of Technology, Cambridge, MA 02139, USA
[3]Department of Neuroscience, Physiology and Pharmacology, University College London, Gower Street, London WC1E 6BT, UK
*Correspondence: tmargri@nimr.mrc.ac.uk
http://dx.doi.org/10.1016/j.neuron.2014.08.001
This is an open access article under the CC BY license (http://creativecommons.org/licenses/by/3.0/).

SUMMARY

Sensory computations performed in the neocortex involve layer six (L6) cortico-cortical (CC) and cortico-thalamic (CT) signaling pathways. Developing an understanding of the physiological role of these circuits requires dissection of the functional specificity and connectivity of the underlying individual projection neurons. By combining whole-cell recording from identified L6 principal cells in the mouse primary visual cortex (V1) with modified rabies virus-based input mapping, we have determined the sensory response properties and upstream monosynaptic connectivity of cells mediating the CC or CT pathway. We show that CC-projecting cells encompass a broad spectrum of selectivity to stimulus orientation and are predominantly innervated by deep layer V1 neurons. In contrast, CT-projecting cells are ultrasparse firing, exquisitely tuned to orientation and direction information, and receive long-range input from higher cortical areas. This segregation in function and connectivity indicates that L6 microcircuits route specific contextual and stimulus-related information within and outside the cortical network.

INTRODUCTION

Through development, the wiring of the cortex is refined to receive and establish both local (Callaway, 1998; Fino and Yuste, 2011; Kätzel et al., 2011; Ko et al., 2011; Kozloski et al., 2001; Yoshimura et al., 2005) and long-range (Berezovskii et al., 2011; Salin and Bullier, 1995) projections that convey information for multimodal integration (Iurilli et al., 2012; Mao et al., 2011) and normal cognitive function (Huang et al., 2014; Reis Marques et al., 2014; Zhang et al., 2014). In many sensory cortical areas, the final organization of the network contains reoccurring features that include dedicated cortico-cortical (CC) versus cortico-thalamic (CT) projection pathways formed by the principal cells found in deep layer six (L6) (Kumar and Ohana, 2008; Marx and Feldmeyer, 2013; Pichon et al., 2012; Thomson, 2010; Zhang and Deschênes, 1997). Although the functional importance of these two output pathways is highlighted by their anatomical prominence, their precise physiological role in cortical and cortico-thalamic processing has proven difficult to dissect.

One approach to understanding the function of cortical pathways in general terms has been to chart regional projectivity (Oh et al., 2014) with the view that the resultant wiring diagram may be used as a template for understanding the emergent physiological properties of underlying circuits (Douglas and Martin, 2007; Reid, 2012). On the other hand, while this approach can provide an overview of connection likelihood and strength—both within (Petersen and Sakmann, 2000) and between (Binzegger et al., 2004; Feldmeyer et al., 2013; Oberlaender et al., 2012) cortical layers and regions—such descriptions are often limited by their cellular and functional resolution (Oh et al., 2014). The difficulty in superimposing precisely the function and connectivity of individual elements within the circuit makes it extremely challenging to accurately attribute potential connectivity rules within a functionally heterogeneous population of neurons and prohibits a detailed understanding of network function.

It is well documented that even at a local level, neurons within the same cortical layer can show significant functional heterogeneity (Allman et al., 1985). In visual cortical areas, the diversity of sensory responses of individual neurons is highlighted by their degree of tuning to the orientation (Hofer et al., 2011; Hubel and Wiesel, 1968; Kerlin et al., 2010; Maruyama and Ito, 2013; Nauhaus et al., 2008; Niell and Stryker, 2008), velocity (Priebe et al., 2006; Roth et al., 2012), and direction of the motion of alternating bars of different luminance (gratings) (Allman et al., 1985; Hubel and Wiesel, 1968; Martin and Schröder, 2013; Roth et al., 2012). These functionally diverse populations are also thought to project to, and receive connections from, multiple cortical layers (Angelucci et al., 2002; Bolz and Gilbert, 1986; Olsen et al., 2012; Thomson and Bannister, 2003) forming interlaminar pathways for integration of both local and long-range input (Berezovskii et al., 2011; De Pasquale and Sherman, 2011, 2013; Glickfeld et al., 2013; Hupé et al., 1998; Salin and Bullier, 1995; Schwarz and Bolz, 1991; Wang and Burkhalter, 2007; Xu et al., 2007). Understanding the functional heterogeneity of cortical networks

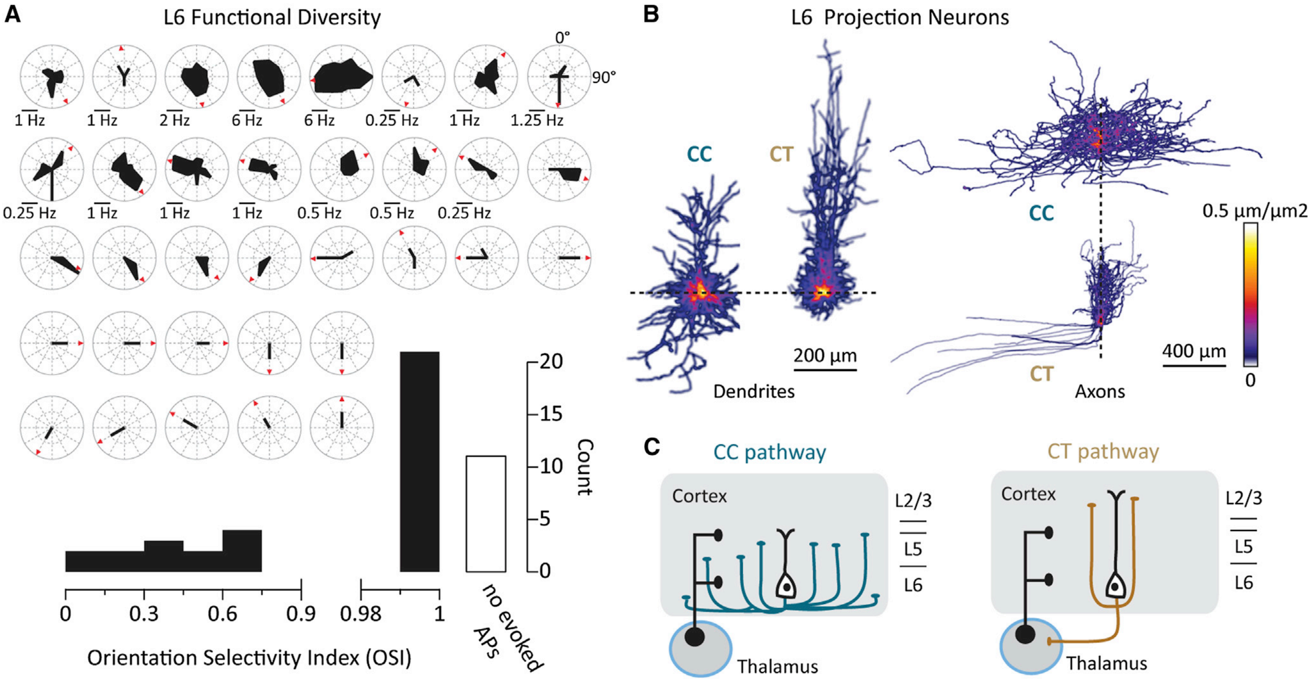

Figure 1. Functional Diversity and Morphologies of V1 L6 Projection Cells

(A) Tuning polar plots for all regular spiking (RS) L6 cells in this study that fired action potentials in response to moving gratings. The strength and tuning of AP firing is indicated by the radial length and orientation of the filled area. Red arrowheads indicate the cells' preferred direction. The scale bar represents 0.25 Hz where not indicated. The histogram shows the population orientation tuning and number of cells that failed to spike during the presentation of any grating; the bin sizes are 0.15 (left) and 0.01 (right).

(B) 3D density projections of the dendrites and axons of neurons separated according to the absence (CC, n = 6) or presence (CT, n = 10) of an axonal thalamic projection.

(C) Schematic of the cortico-cortical and cortico-thalamic pathways morphologically defined by the axonal projection of these two classes of principal neurons.

therefore requires simultaneous analysis of their cellular composition (Helmstaedter et al., 2013; Oberlaender et al., 2012), sensory response properties (Niell and Stryker, 2008; Oberlaender et al., 2012), input connectivity, and output projectivity (Briggman et al., 2011; Denk et al., 2012).

Here in mouse V1 we have undertaken an in vivo single-cell analysis of the sensory response properties and connectivity of the L6 network that is known to contain a functionally heterogeneous population of principal cells (Hirsch et al., 1998; Niell and Stryker, 2008) that comprise CC- and CT-projecting neurons (Briggs, 2010; Katz, 1987; Kumar and Ohana, 2008; Marx and Feldmeyer, 2013; Pichon et al., 2012; Thomson, 2010; Usrey and Fitzpatrick, 1996; Zhang and Deschênes, 1997). By targeting retrograde transsynaptic tracing (Marshel et al., 2010; Rancz et al., 2011; Wickersham et al., 2007) to individually recorded cells (Rancz et al., 2011) and charting their brain-wide connectivity, we find that CC- versus CT-projecting neurons relay functionally distinct signals and are differentially innervated by higher-order cortical areas.

RESULTS

We performed blind in vivo whole-cell recordings (Margrie et al., 2002) in V1 of anesthetized mice at a depth of 600 to 950 μm from the pial surface (n = 81 cells). On the basis of their recorded intrinsic properties including the initial action potential (AP) half-width, the mean frequency of firing, and input resistance, we could distinguish fast spiking cells from regular spiking (RS) neurons (Figure S1 available online). These criteria were used to identify the RS population of L6 cells (n = 74) expected to mediate the CC and CT pathways under investigation in this study. To begin to explore the functional diversity of L6 principal cells, we first recorded the AP tuning of RS neurons in response to moving sinusoidal gratings (Figure 1A). The stimulus-evoked instantaneous firing rate of RS cells extended over a large range (0–400 Hz) and was found to encompass a broad range of selectivity to the orientation and direction of the gratings (Figure 1A).

Classification of L6 Principal Cells

In order to attribute these diverse response properties to specific types of L6 projection neurons, we performed morphological reconstructions (n = 16 cells) and identified two distinct anatomical classes (Marx and Feldmeyer, 2013; Thomson, 2010; Zhang and Deschênes, 1997) (Figures S2A–S2E). The first group (n = 6) exhibited a large dendritic field (convex envelope = 0.0077 ± 0.0011 mm^3) and an elongated total basal dendritic length (1,884 ± 303 μm) with dendrites rarely extending beyond layer 4 (Figure S2B; Figure 1B). The dendritic tree of this neuronal class was morphologically diverse, displaying classical upright, but also inverted and tangential projecting apical dendrites

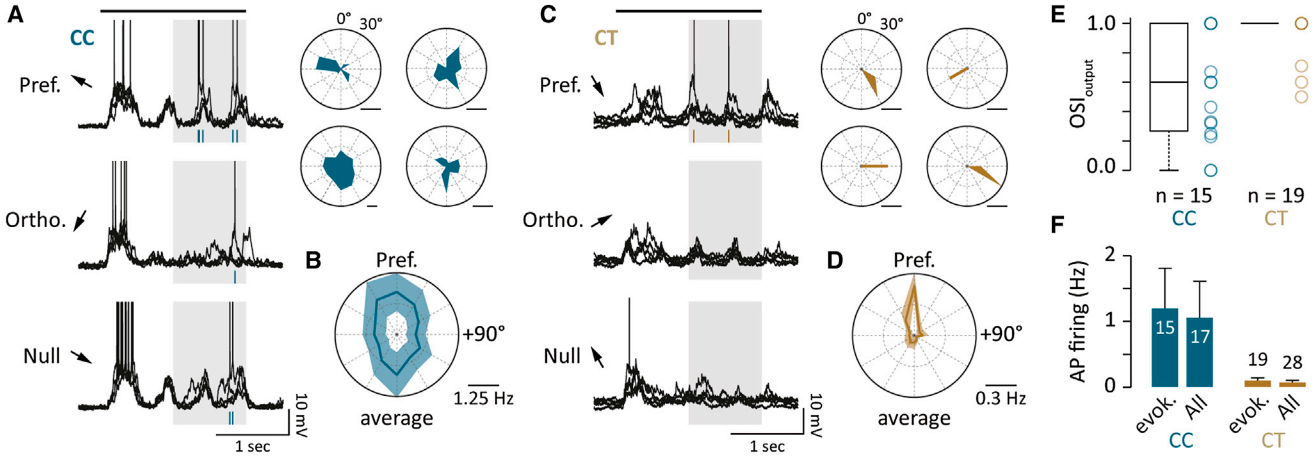

Figure 2. Orientation-Dependent AP Tuning in CC and CT Neurons

(A) Left: examples of four membrane voltage traces of spiking responses to gratings moving in the preferred, orthogonal, and antipreferred (null) directions for a CC cell. Spikes are represented as raster ticks under the traces. The black bar indicates the stimulus motion, and the shaded area indicates the analysis time window. Right: polar plots are shown from four representative CC cells). The polar plot corresponding to the example cell is shown (top left).

(B) Average polar plot from all CC cells that fired spikes in response to moving gratings aligned to preferred direction.

(C) Left: example of five membrane voltage traces of spiking responses to gratings moving in the preferred, orthogonal, and antipreferred (null) directions for a CT cell. Spikes are represented as raster ticks under the traces. The shaded area indicates the analysis time window. Right: polar plots from four representative CT cells are shown. The polar plot corresponding to the example cell is shown (top left).

(D) Average polar plot from all CT cells that fired spikes in response to moving gratings aligned to preferred direction.

(E) Box plot of AP orientation selectivity scores for all CC and CT cells exhibiting evoked firing.

(F) Bar graphs of evoked mean AP firing rates for CT and CC neurons excluding and including those cells in which no evoked APs were observed.

Error bars show SEM.

(Figure S2B). Their axonal morphology was strikingly elaborate (total length 14,152 ± 2,666 μm; Figure 1B), with an extensive cortical horizontal span (1,070 ± 223 μm; Figure S2E) largely contained within layers 5 and 6 (13% ± 4% and 73% ± 4% of total length, respectively; Figures S2B and S2E). Arborizations often entered the white matter and returned to the cortex and regularly extended into secondary visual areas. These dendritic and axonal morphological properties are consistent with previous anatomical descriptions of CC-projecting L6 cells (Figure 1C) (Pichon et al., 2012; Zhang and Deschênes, 1997).

The second group contained cells that extended their apical dendrites beyond the L4-L5 border (n = 10) and, despite having a similar total dendritic length (CC: 4,038 ± 1,090 μm versus 3,297 ± 738 μm [SD], $p > 0.05$), exhibited a significantly less elaborate dendritic morphology (Figures S2C and S2D; Figure 1B) with a smaller convex envelope (0.0046 ± 0.0004 mm^3, $p < 0.01$) and a shorter total basal dendritic length (1,126 ± 74 μm, $p < 0.01$; Figures S2C and S2D; Movie S1). For this class, dendrites exhibited a significantly higher branching frequency compared to the CC cells (0.91 ± 0.06 versus CC = 0.61 ± 0.05 nodes/100 μm, $p < 0.01$) and had a lower mean spine density (0.52 ± 0.02 [n = 4 cells] versus CC: 0.64 ± 0.06 [n = 3 cells] spines/μm, $p < 0.05$; Figure S2D). Despite reconstructing the axons of this cell type beyond the cortical boundaries used for the CC analysis, axons appeared less elaborate (Figures 1B, S2C, and S2E), with a reduced total length (4,226 ± 519 μm; $p < 0.01$) and cortical horizontal span compared to the CC cells (299 ± 22 μm, $p < 0.001$; Figure S2E). Within the cortex, the axons of this group rarely extended beyond the medial and lateral dendritic boundaries and projected vertically at least to layer 5 (Movie S2). All of the cells in this morphological class projected one axonal branch into the white matter and then turned laterally into the thalamic tract, reminiscent of L6 CT-projecting pyramidal cells (Kumar and Ohana, 2008; Pichon et al., 2012; Zhang and Deschênes, 1997) (Figure 1C).

Inspection of the intrinsic properties of the reconstructed neurons revealed several biophysical features (Brumberg et al., 2003; Kumar and Ohana, 2008) (initial instantaneous AP firing rate, early accommodation of firing rates, slope of the relationship between the evoked AP firing frequency (F1) and the amplitude of underlying injected current (I), the amount of hyperpolarization-evoked membrane potential depolarization [membrane sag]; see Figures S3A and S3B) that may be used to electrophysiologically distinguish between regular spiking neurons according to their axonal projectivity. Using only these intrinsic parameters, we performed a cluster analysis on all regular spiking cells (n = 74 cells; Figure S3C) that produced two main clusters, whereby one group contained all of the morphologically confirmed CC neurons and the other contained the CT-projecting cells. These intrinsic parameters therefore reflect biophysical regularities of the CC and CT populations and were used to assign all L6 regular spiking cells recorded in this study (Figure S3C).

Orientation Tuning in CC and CT Cells

Following this classification procedure, we could determine that AP firing in CC cells was broadly tuned (Figure 2A) since spikes could be evoked by gratings moving in several directions and

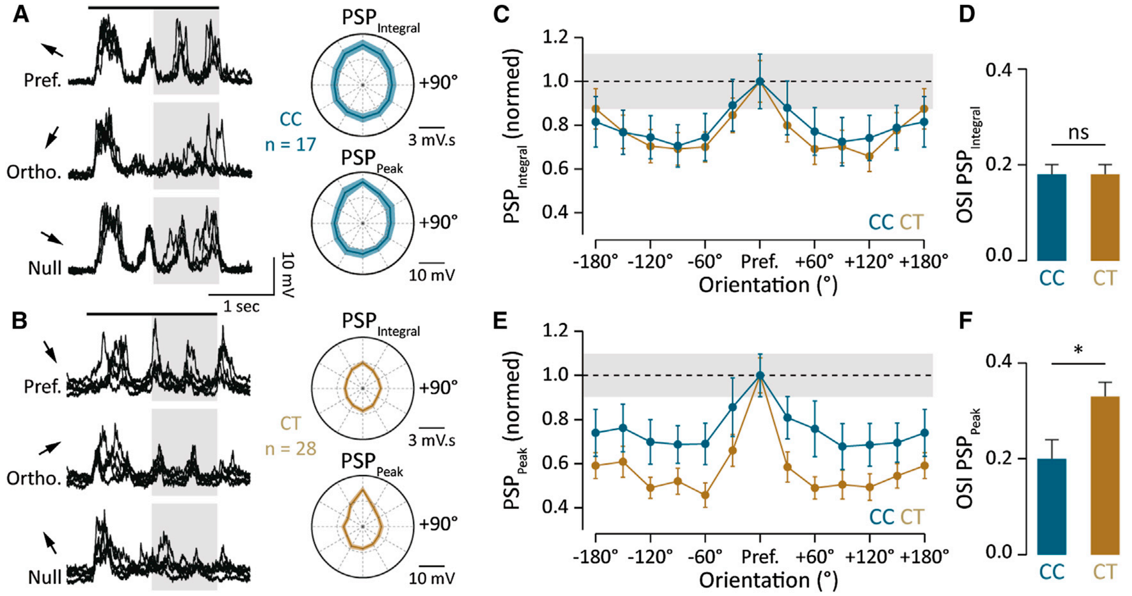

Figure 3. Orientation-Dependent Synaptic Tuning in CC and CT Neurons

(A) Left: example of four membrane voltage recordings of the synaptic response to gratings moving in the preferred, orthogonal, and antipreferred (null) directions for a CC cell (spikes are clipped). The black bar indicates the stimulus motion, and the shaded area indicates the analysis time window. Right: CC population polar plots show the tuning of the mean PSP integral (top) and peak amplitude (bottom) for all orientations.

(B) Left: example of five membrane voltage recordings of the synaptic response to gratings moving in the preferred, orthogonal, and antipreferred (null) directions for a CT cell (spikes are clipped). The shaded area indicates the analysis time window. Right: CT population polar plots show the tuning of the mean PSP integral (top) and peak amplitude (bottom) for all orientations.

(C) Normalized tuning plot comparing the integral of the PSP depolarization for CC and CT cells for each grating orientation. The shaded line indicates the standard error of the mean of the CC population at the preferred direction.

(D) Orientation selectivity index scores of the integral of the evoked PSP (OSI $PSP_{integral}$) for CC and CT cells.

(E) Normalized tuning plot directly comparing the peak amplitude of the PSP depolarization for CC and CT cells for each grating orientation. The shaded line indicates the standard error of the mean of the CC population at the preferred direction.

(F) Orientation selectivity index scores of the evoked PSP peak (OSI PSP_{peak}) for CC and CT cells.

Error bars show SEM.

typically included the orientation orthogonal to the preferred (Figures 2A and 2B). In stark contrast, the firing of CT cells was highly orientation tuned, predominantly firing in only one direction (OSI_{output} CC [n = 15]: median = 0.6, Q1 = 0.25, Q3 = 1; CT [n = 19]: median = 1, Q1 = 1, Q3 = 1, $p < 0.01$; Figures 1A and 2C–2E). While this cluster-analysis-based classification of cell function reveals differences in tuning and that specific, highly precise direction signals are conveyed to thalamic targets, it also indicates that CC cells have a high overall probability of evoking APs (Figure 2F). This may suggest that differences in cell excitability could explain poor tuning in CC cells that would be consistent with the lower rheobase (Kumar and Ohana, 2008) and initial AP bursting properties (Kumar and Ohana, 2008; Ledergerber and Larkum, 2010) observed for this population (Figures S3A and S3B).

We therefore next sought to establish the extent to which the underlying evoked input could account for the broad versus narrow range of AP tuning profiles observed in CC and CT cells, respectively. We first quantified the integral of the evoked postsynaptic potential (PSP) and determined that CC cells receive significantly more net depolarization than CT cells (CC = 3.8 ± 0.5 mV.s [n = 17] versus CT = 2.3 ± 0.2 mV.s [n = 28], $p < 0.01$; Figures 3A and 3B). However, the relative amount of depolarization per grating stimulus revealed little preference for its orientation (OSI $PSP_{Integral}$: CC = 0.18 ± 0.02 versus CT = 0.18 ± 0.02, $p > 0.9$; Figures 3C and 3D). On the other hand, for both cell types the PSP peak amplitude (Figures 3A and 3B) displayed a significant preference for grating orientation (OSI L6 $PSP_{Integral}$ = 0.18 ± 0.01 versus PSP_{Peak} = 0.28 ± 0.03, $p < 0.01$) and direction (DSI L6 $PSP_{Integral}$ = 0.1 ± 0.02 versus PSP_{Peak} = 0.23 ± 0.03, $p < 0.01$). This improved tuning of the PSP peak was most striking for CT cells (OSI: CT PSP_{Peak} = 0.33 ± 0.03 versus CT $PSP_{Integral}$ = 0.18 ± 0.02, $p < 0.01$; Figures 3E and 3F) such that already for gratings presented at 30° from the preferred direction, the average amplitude of the peak depolarization was significantly reduced (PSP_{Peak}: Pref. = 14.6 ± 1.2 mV versus ± 30° = 9.1 ± 1 mV, n = 28, $p < 0.01$; Figure 3E). Compared to its integral, the peak amplitude of the PSP therefore conveys the most accurate orientation and direction information, whereby the inputs onto CT cells are the most strongly tuned (OSI PSP_{Peak}: CC = 0.2 ± 0.04 versus CT = 0.33 ± 0.03, $p < 0.01$; Figure 3F). This indicates that the CC population receives comparatively strong yet broadly tuned synaptic drive, while the CT cells receive a highly tuned orientation signal.

Although CT cells show sharp synaptic and AP tuning, the orientation selectivity of the input may not necessarily cause

CellPress

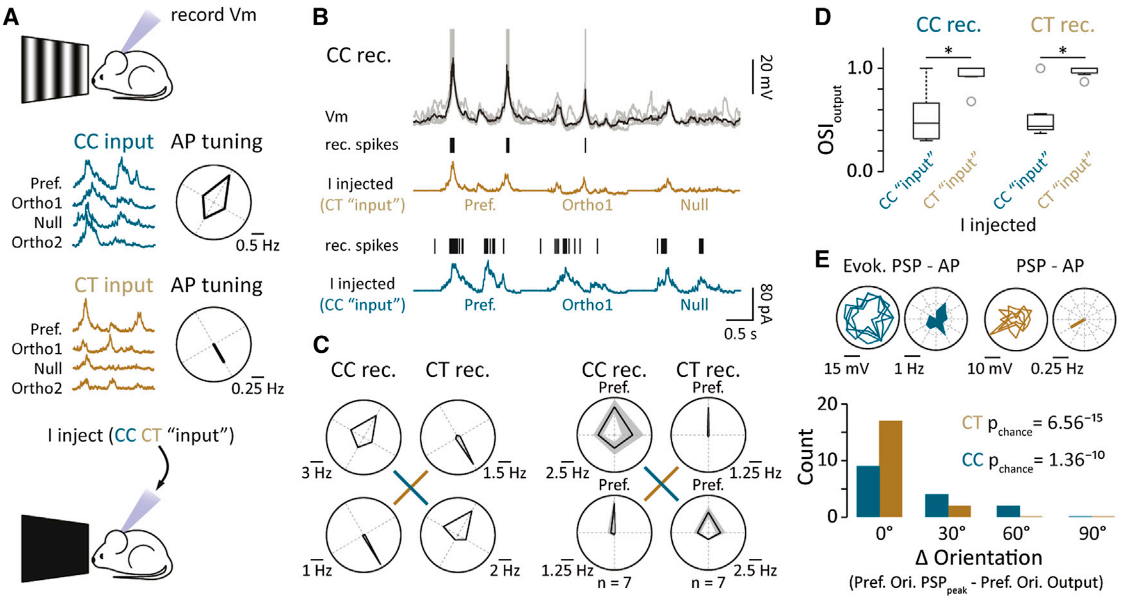

Figure 4. Output Tuning Is Independent of Biophysical Properties

(A) Schematic showing the design for experiments performed in (B)–(D). Individual membrane potential traces recorded in response to drifting gratings for the preferred and related cardinal directions recorded in a CC (blue) and a CT (brown) cell. Polar plots show the mean AP tuning for the same cardinal directions in the same two cells.

(B) Average and five individual membrane potential traces recorded in a CC cell during injection of the CT PSP waveform (brown). Spikes recorded in response to the injected waveforms are indicated by the raster plot (black). An example of the injected CC PSP waveform (blue) and resultant spikes is recorded in the same CC cell.

(C) Left: polar plots for an example CC and CT cell in which the injected waveforms are the same as shown in (A). These polar plots may be directly compared to (A). Right: population polar plots for comparing injections of CC and CT responses into either CC or CT cells are shown. Three different sets of injection waveforms were used.

(D) Box plot showing the range of orientation selectivity index scores for all injected cells. Plots are aligned to the preferred orientation of the AP output of the cells from which the injected waveforms were obtained.

(E) Top: example polar plots from a CC (blue) and CT (brown) cell showing the tuning of the peak amplitude of the PSP and AP. PSP polar plots are displaying four repetitions of each stimulus. AP polar plots are showing the mean firing rate for each repetition. Bottom: a histogram of the difference in the orientation preference of the PSP_{peak} versus the AP response in all CC and CT cells.

Error bars show SEM.

the apparent exquisite tuning of AP output. CT cells are extremely sparse firing (CC = 1.2 ± 0.61 Hz versus CT = 0.1 ± 0.04 Hz, $p < 0.05$; Figure 2F). For example, over multiple stimulus repetitions CTs may only discharge one or two APs. Such sparse spiking may therefore occur spontaneously, independent of the stimulus. We therefore looked to establish causality in PSP-AP tuning by injecting PSP waveforms evoked in both CC and CT cells (Figure 4A) back into individual neurons in the absence of visual stimulation (Figures 4A and 4B). We found that the injected PSPs faithfully reproduced the grating-evoked CC and CT cell AP tuning, irrespective of the identity of the injected neuron (Figures 4B and 4C). Thus, the AP tuning of these two groups (Figures 4C and 4D) results directly from the cell-type-specific dynamics of the evoked PSP (CC: CC_{inject} median = 0.47, Q1 = 0.31, Q3 = 0.69 versus CT_{inject} median = 1, Q1 = 0.92, Q3 = 1, n = 7, $p < 0.05$; CT: CC_{inject} median = 0.44, Q1 = 0.4, Q3 = 0.56 versus CT_{inject} median = 1, Q1 = 0.94, Q3 = 1, n = 7, $p < 0.05$; Figure 4D). Consistent with these data, we also find that the specificity of the grating-evoked CT spiking is a highly reliable indicator of the orientation preference of the underlying synaptic input (Figure 4E). Synaptic signaling onto L6 principal cells therefore produces two functional distinct distributions of tuning profiles, whereby sparse, highly orientation-selective information is relayed to thalamic target areas.

Connectivity onto CC and CT Cells

These data show that across the population, different morphological classes of L6 cells relay specific visual information and form unique signaling pathways within and outside the V1 circuit. One might therefore expect these functionally discrete CC and CT populations to be targeted by specific upstream pathways. To begin examining the functional specificity of CC and CT connectivity, we targeted retrograde transsynaptic tracing from individually recorded neurons using a glycoprotein deficient form of the rabies virus encapsulated with the avian sarcoma and leucosis virus envelope protein (ΔRV) (Wickersham et al., 2007). By performing whole-cell recordings with internal solutions containing DNA vectors (Rancz et al., 2011), we could drive the expression of the envelope protein receptor (TVA) and the RV glycoprotein (RVG) that are required for single-cell targeted infection and monosynaptic retrograde spread of ΔRV (Figure 5A). Immediately following whole-cell

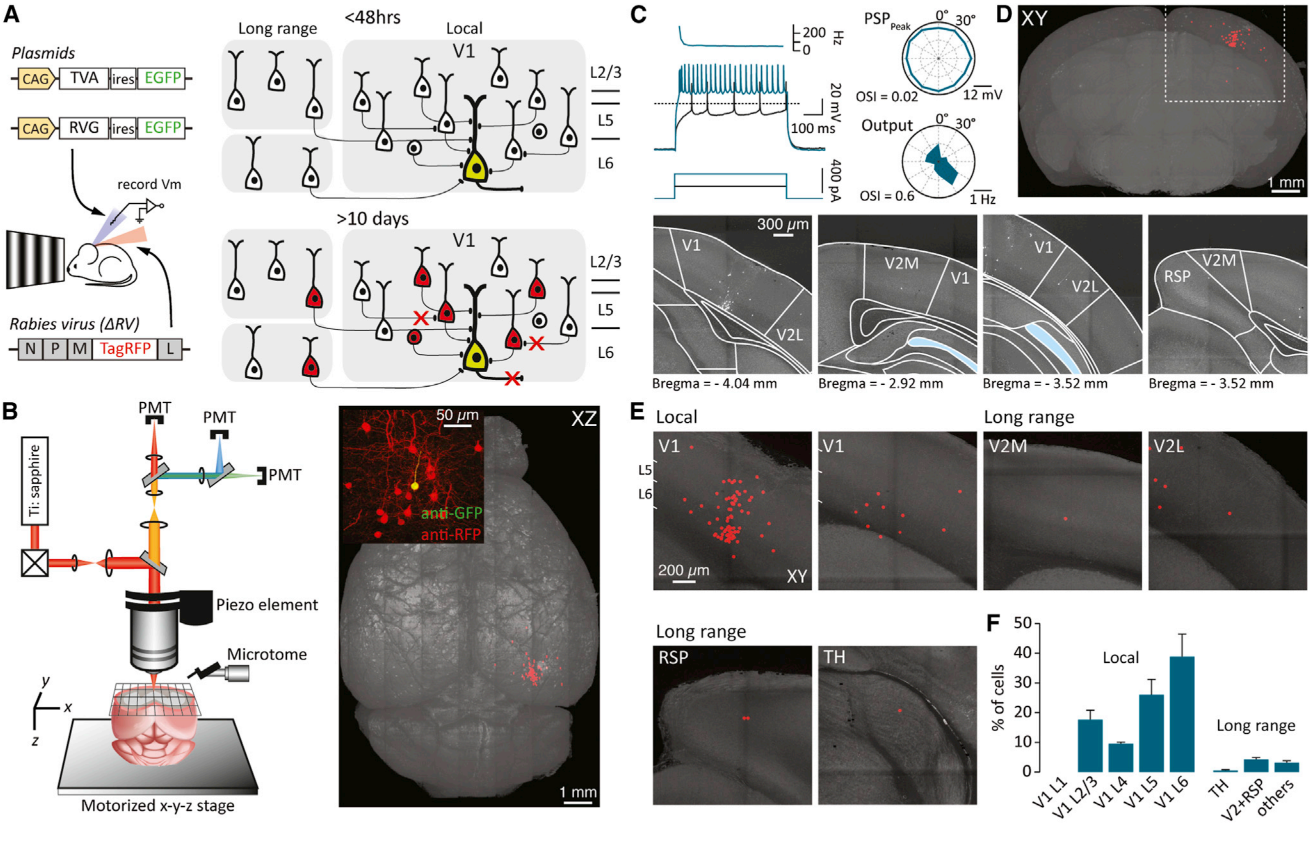

Figure 5. Mapping Connectivity onto Individual CC Cells

(A) During whole-cell recording, the cell was loaded with DNA plasmids to drive expression of the rabies glycoprotein (RVG) and the avian virus receptor (TVA). This was followed by injection of the modified rabies virus (ΔRV) into the local area that results in targeted infection of the recorded neuron and subsequent retrograde spread and expression of RV-RFP.

(B) After at least 10 days postrecording, the brain was fixed and placed under a serial two-photon microscope (left) for whole-brain serial imaging. Inset: a coronal postimmunostained confocal image of the recorded (yellow) and local presynaptic cells (red) is shown.

(C) Left: membrane-voltage traces recorded at and two times the rheobase. Top left: the instantaneous frequency of AP firing at two times the rheobase is shown. Right: tuning polar plots of the same CC cell recorded during delivery of plasmids for RV targeting and tracing.

(D) Top: coronal two-photon whole-brain image stack showing the location of cells labeled with the modified rabies virus following electrophysiological characterization of the recorded cell in (C). Bottom: following imaging, the labeled cells were localized using a standard mouse brain atlas. Regions relevant to this study include the primary visual cortex (V1), the medial and lateral secondary visual cortices (V2M and V2L, respectively), the retrosplenial cortex (RSP), and the thalamus (TH).

(E) Example coronal images of the marked location of labeled cells (red spheres) within V1 (local) and outside V1 (long range).

(F) Histogram showing the relative distribution of labeled cells (n = 3 mice).

Error bars show SEM.

recording with the plasmid-containing internal solution, we injected ΔRV and up to 12 days later performed whole-brain serial two-photon tomography (Osten and Margrie, 2013; Ragan et al., 2012) (Figure 5B) to chart the spatial profile of presynaptic cells.

To directly assess the regularity of connectivity onto these two classes of L6 neurons, the intrinsic, synaptic, and AP tuning response properties were first recorded (Figure 5C). Following singe-cell rabies tracing, we found that for CC cells more than 90% of the labeled presynaptic neurons (138 ± 21 labeled cells, n = 3; Figures 5D and 5E; Movie S3) were located locally within V1. The majority of these presynaptic cells were observed in layers 5 (26% ± 5.2%) and 6 (38.9% ± 7.6%) although input from layer 2/3 (17.6% ± 3.3%) and to a lesser extent layer 4 (9.6% ± 0.5%) was also apparent (Figures 5E and 5F). Very few long-range projecting cells were observed, though a small fraction of presynaptic neurons were found in areas including thalamus (0.5% ± 0.3%), secondary visual (2.9% ± 0.7%), and retrosplenial (1.4% ± 0.4%) cortices.

Single-cell rabies tracing in CT cells revealed almost three times the number of presynaptic cells when compared to CC neurons (383 ± 70 labeled cells, n = 4; Figures 6A and 6B; Movie S3). In relative terms, CT cells received almost the identical fraction of inputs from layer 2/3 (18.8% ± 3.7%) and layer 4 (10.7% ± 1.7%) within V1 as that observed for CC cells (Figures 6C and 6D). In contrast, CT neurons received more than 20% of input from secondary visual and retrosplenial cortices (V2 + RSP: CC = 4.2% ± 0.7% versus CT 20.8% ± 5.2%,

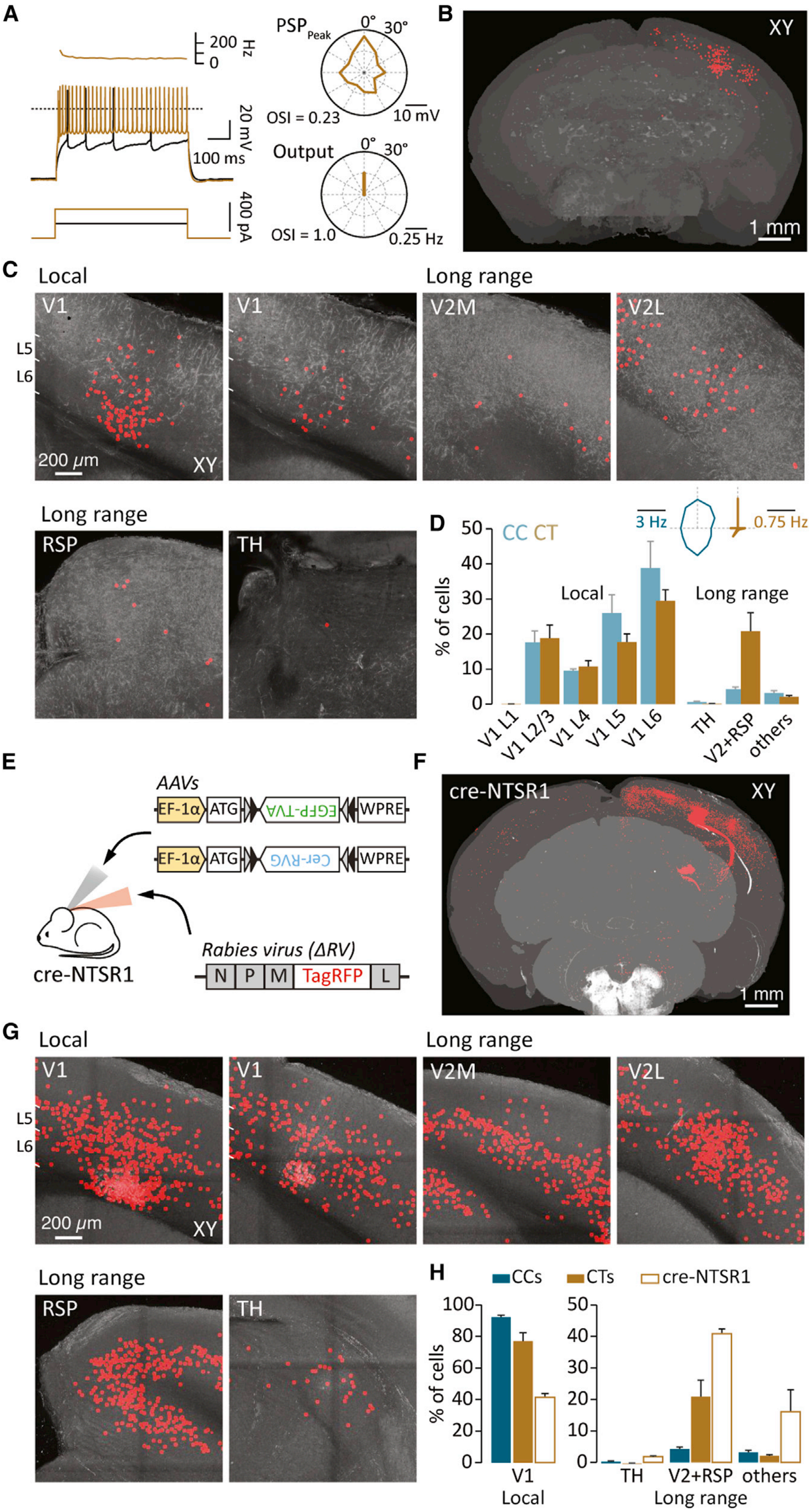

Figure 6. Connectivity Maps of CT and NTSR1-Expressing Cells

(A) Left: membrane-voltage traces recorded at and two times the rheobase. Top left: the instantaneous frequency of AP firing at two times the rheobase is shown. Right: tuning polar plots of a CT cell recorded while delivering plasmids for RV targeting and tracing are shown.

(B) Coronal projection of a two-photon whole-brain image stack showing the location of cells labeled with the modified rabies virus following electrophysiological characterization of the recorded cell in (A).

(C) Example coronal images of the location of labeled cells within V1 (local) and outside V1 (long range).

(D) Histogram showing the relative distribution of labeled cells for CT and CC cells (n = 4 and 3 mice, respectively). Inset: the average tuning profile (aligned to the preferred direction) of the recorded host cells in which single-cell rabies tracing was performed is shown.

(E) Schematic showing the experimental design whereby a cre-dependent AAV is injected into cre-NTSR1^{+ve} mice for targeted RV infection of CT cells.

(F) Two-photon whole-brain image stack showing the location of labeled presynaptic cells. In this brain, 421 putative host cells (not shown) were all located in L6 within V1.

(G) Example two-photon images showing local and long-range connectivity onto the NTSR1^{+ve} cell population.

(H) Histograms showing the fraction of presynaptic cells located within and outside V1 for CC, CT, and cre-NTSR1^{+ve} cells.

Error bars show SEM.

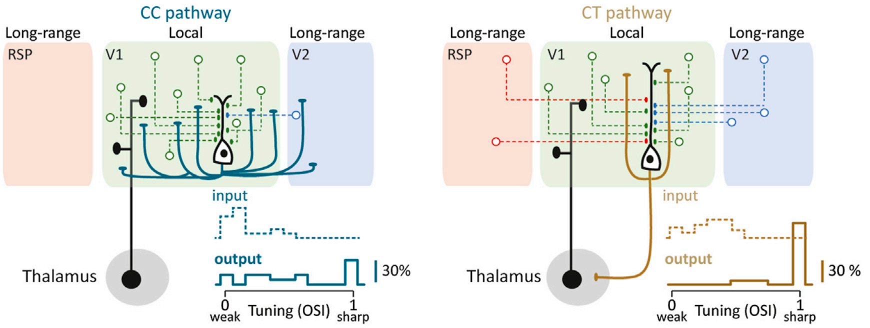

Figure 7. Functional Specificity and Connectivity of CC and CT Pathways

Left: schematic showing CC-projecting cells receiving inputs from neurons located primarily within V1. On average, CC cells receive weakly tuned synaptic input and show poorly tuned output firing. Inset: a population histogram of PSP_{peak} (input, dashed line, n = 17) and AP (output) tuning (n = 15) for all CC cells recorded in this study (bin size = 0.1) is shown. Right: a schematic showing CT-projecting cells receiving comparatively more long-range inputs from neurons located in V2 and RSP is shown. Inset: a population histogram of PSP_{peak} (input, dashed line, n = 28) and AP (output) tuning (n = 19) for all CT cells recorded in this study (bin size = 0.1) is shown.

$p < 0.05$; Figures 6C and 6D). Consequently, CT cells had relatively fewer input cells located in deeper layers 5 and 6 of V1 (L5 = 17.7% ± 2.4% and L6 = 29.5% ± 3.2%; Figure 6D).

Our electrophysiological and input tracing data indicate that the upstream network connectivity of individual L6 neurons is not random but respects the morphological and hence the functional identity of the target neuron. If these single-cell-based connectivity maps highlight general rules of monosynaptic connectivity onto these two classes of neurons, one might expect genetically targeted input tracing onto a specific population to show similar connectivity profiles. To examine the correspondence between our single CT cell-based maps and the broader L6 CT population, we took advantage of the fact that in L6 of mouse V1, CT-projecting cells are known to selectively express neurotensin receptor 1 (NTSR1) (Gong et al., 2007). By using a cre-NTSR1^{+ve} mouse line, we targeted injections of cre-dependent AAV helper viruses to drive expression of the RV glycoprotein and the avian receptor protein across the CT population (n = 4; Figures 6E and 6F). When comparing to CC cells, presynaptic connectivity in cre-NTSR1^{+ve} mice was widespread (Figure 6G), with relatively fewer cells in V1 (CC = 92.1% ± 1.2% versus cre-NTSR1 = 41.3% ± 2.4%, $p < 0.01$) and substantially more labeled neurons located in secondary visual and retrosplenial cortices (V2 + RSP CC = 4.2% ± 0.7% versus cre-NTSR1 = 40.8% ± 1.5%, $p < 0.01$, CT = 20.8% ± 5.2%, $p < 0.05$; Figures 6G and 6H).

Taken together, despite receiving most of their synaptic drive from neurons located within the V1 circuit, the L6 CC pathway conveys visual motion signals that cover a broad spectrum of orientation selectivity (Figure 7). On the other hand, cells relaying motion information to thalamus output exquisitely tuned orientation- and direction-related signals and received substantial widespread innervation from higher-order cortical areas known to convey visual and spatial information (Figure 7).

DISCUSSION

This study shows that in V1, the functional diversity of L6 can be attributed to specific populations of output neurons that are embedded in different anatomical microcircuits. We show that the output tuning of CC and CT cells can be directly attributed to the tuning profile of the somatic depolarization, rather than to their difference in intrinsic properties. At least for the stimuli used here, the CC and CT cell populations are wired to receive functionally distinct, direction-related synaptic signals and therefore appear to play distinct roles in visual processing.

We reveal that several intrinsic biophysical parameters of L6 neurons recorded in vivo may be used to classify cells according to their morphological identity and projection targets. Although we have not directly determined the differential impact of these properties on the integration of signals in the dendrites of CC and CT neurons, it seems likely they will impact PSPs arriving at the soma (Chadderton et al., 2014). Experiments in which we injected previously recorded visually evoked somatic responses back into CC and CT cells indicate that their output tuning cannot simply be explained by differential intrinsic membrane properties expressed proximal to the axonal initial segment or soma.

Morphological studies in V1 of some species indicate three classes of principal L6 neurons. For example, on the basis of their dendritic profile, claustrum-projecting neurons in cat have morphological features similar to that of CT-projecting cells described here (Katz, 1987). However, previous studies injecting retrograde tracers into the claustrum (Carey and Neal, 1985) indicate that in rat, claustrum-projecting cells in the visual cortex are confined to the deep layers of secondary visual areas. The axonal projection from our population labeling experiments indicated that cre-NTSR1^{+ve} L6 cells in mouse V1 do not target the claustrum. Also, on the basis of our biocytin reconstructions of CC cell axons, we find no evidence for a direct claustrum projection. However, since CC cells in V1 can extend their axons into deep layers of secondary visual areas, an indirect primary visual cortical-claustrum pathway may exist.

Our data show that the broad stimulus selectivity in L6 can be attributed specifically to the CC-projecting population. These cells receive more net depolarization during drifting gratings, yet they are, on average, only modestly tuned to stimulus orientation. In contrast, CT neurons were more selective for stimulus orientation and/or direction. Since we have not directly determined the functional identity of the presynaptic cells providing visual information, the precise contribution of intra- versus interlaminar connectivity to the subthreshold tuning of CC and CT neurons remains to be established. In V1, subsets of GABA-ergic interneurons also exhibit broad stimulus selectivity (Sohya et al., 2007; Kerlin et al., 2010; Hofer et al., 2011; Niell and Stryker,

2008). Their tuning is believed to arise from them receiving input from many local pyramidal cells tuned to different stimulus orientations (Chen et al., 2013; Hofer et al., 2011; Bock et al., 2011). Our data indicate that CC cells receive substantial synaptic input from within L5 and L6 (Mercer et al., 2005; Zarrinpar and Callaway, 2006) and raise the possibility that such rules of local functional convergence are not specific to inhibitory interneurons but may also apply to this principal cell type. It also remains a possibility that multiple subclasses of CC cells—differentially tuned to orientation and/or direction—receive input from nonoverlapping constellations of presynaptic cells.

The paucity of information about the influence of cortex on thalamus arises, at least in part, from the functional and morphological diversity of L6 neurons. Compared to CC cells, our data show that synaptic drive onto CT cells is highly selective for stimulus orientation and/or direction and that CT cell firing is extremely sparse. The fact that CT axons project to thalamic structures, including the dorsal lateral geniculate nucleus and the reticular nucleus, indicates that this highly selective feedback signal may be used for stimulus-specific thalamic gain control via excitatory and inhibitory modulation (Mease et al., 2014; Olsen et al., 2012). This highly tuned CT output signal is also expected to directly impact activity within layers 5 and 6 and, via ascending polysynaptic pathways, provide modulation of upper cortical layer activity (Bortone et al., 2014; Olsen et al., 2012).

The observation that CC and CT cells have a similar total dendritic length (Oberlaender et al., 2012) and that CTs have a lower spine density appears at odds with our observation that CT cells receive input from more than double the number of presynaptic cells. This may suggest that the number of contacts per connection is reduced in CT cells such that CTs are more densely innervated (in terms of the number of presynaptic cells) compared to CC cells. Alternatively, it is possible that cell-specific tropisms may impact or bias the retrograde transmission of the rabies virus in some way. However, the fact that we find presynaptic cells located in distant areas, including the thalamus, indicates that long-range inputs can be labeled in both cell types. The increased connectivity onto CT cells may therefore reflect a dynamic role in integration of information converging from across functionally nonoverlapping upstream networks. Dense innervation of CT cells may also be explained by them receiving input from a larger number of inhibitory neurons that ensure the sharpening of CT responses (Chadderton et al., 2014).

While our study does not attempt to explain the relative contribution of presynaptic neurons to stimulus selectivity in L6 principal cells, these data do however show that the sparsely encoded information about stimulus direction is processed by a specific subpopulation of L6 neurons that are biased in receiving input from secondary visual and retrosplenial cortices. This L6 pathway may therefore mediate thalamic integration of both cortical visual and egocentric information. Integration of self-motion and head-direction signals (Clark et al., 2010) within V1 L6 CT cells could optimize object motion detection (Hupé et al., 1998) by providing a contextual influence on thalamic relay neurons.

Approaches that combine physiological analysis with dense electron microscopy-based reconstruction are elucidating the circuit organization mediating stimulus motion processing at the very early stages of the visual system (Briggman et al., 2011; Helmstaedter et al., 2013). Establishing the relation between function and connectivity in large-scale cortical circuits however remains exceedingly challenging (Bock et al., 2011; Reid, 2012). The combination of in vivo single-cell physiology and retrograde monosynaptic tracing enables identification of local and global projections onto individual cells whose subthreshold sensory response properties have been characterized, an approach that permits the generation of cortical wiring diagrams with single-cell and functional resolution. As recently shown in other sensory systems (Angelo et al., 2012), here we find that L6 CC- versus CT-projecting cells have distinct intrinsic and functional properties. Furthermore, these two classes of projection cells are embedded within distinct wiring motifs that indicates top-down, targeted innervation of L6 microcircuits may provide contextual modulation during sensory computation.

EXPERIMENTAL PROCEDURES

In Vivo Recordings and Visual Stimulation

Surgical Procedures

Adult C57/BL6 mice (5–8 weeks old) were anaesthetized with a mixture of Fentanyl (0.05 mg/kg), Midazolam (5.0 mg/kg), and Medetomidin (0.5 mg/kg) in saline solution (0.9%; intraperitoneal) and supplemented as necessary (20% of initial dose). Mice were head fixed using nonpuncture ear bars and a nose clamp (SG-4N, Narishige, Japan), and their body temperature was maintained at 37°C –38°C using a rectal probe and a heating blanket (FHC, Bowdoinham, ME, USA). An incision was made in the scalp and a small craniotomy was drilled above the primary visual area of the cortex using a dental drill (Osada Electric, Japan) and the dura removed. Following recordings, the craniotomy was sealed using a silicone sealant (Kwik-Sil, World Precision Instruments) and the scalp sutured. Anesthesia was reversed by injection of a mixture of Naxolon (1.2 mg/kg), Flumazenil (0.5 mg/kg), and Atipamezol (2.5 mg/kg) in saline solution (0.9%). The wound was infiltrated with lidocaine and an antibiotic (Cicatrin, GlaxoSmithKline, UK) topically applied. During initial recovery, mice were kept in a climate-controlled chamber (Harvard Apparatus, Holliston, MA, USA) for 3–4 hr under observation. All procedures were approved by the local ethics panel and the UK Home Office under the Animals (Scientific Procedures) Act 1986.

Whole-Cell Recordings

In vivo whole-cell recordings were carried out as described previously (Margrie et al., 2002) using a Mulitclamp 700B amplifier (Axon Instruments, USA). Data were filtered at 4 KHz and digitized at 10–20 kHz using an ITC-18 A/D-D/A interface (InstruTECH, Heka Elektronik, Germany) and the Neuromatic package (http://www.neuromatic.thinkrandom.com) under Igor Pro 5 (http://www.wavemetrics.com). Intracellular solutions for whole-cell recordings were made up in concentrated stock (two times the final concentration) to allow for biocytin or plasmid addition. The final concentrations were (all from Sigma-Aldrich or VWR International, UK) 110 mM K-methanesulphonate, 40 mM HEPES, 6 mM NaCl, 0.02 mM $CaCl_2$, 3 mM $MgCl_2$, 0.05 mM EGTA, 2 mM Na_2ATP, 2 mM MgATP, and 0.5 mM Na_2GTP; the pH was adjusted to 7.28 using KOH. The final osmolarity after adding either 0.5% biocytin or suspended plasmids was adjusted to the range of 280–294 mOsm. Intracellular solutions were filtered through a 0.22 μm pore size centrifuge filter (Costar Spin-X). Plasmid concentrations were verified by spectrophotometry (NanoDrop 2000, Thermo Scientific): 200 ng/μl RVG plasmid and 40 ng/μl TVA plasmid. In some cases an XIAP plasmid (40 ng/μl) was also included. For individual CC and CT tracing experiments, only one whole-cell recording was performed in each brain within a maximum of three attempts. Information relating to plasmid and virus production is provided in the Supplemental Information.

Visual Stimulation

Visual stimuli were generated using MATLAB (MathWorks) and the Psychophysics Toolbox. Stimuli were presented on a 56 cm LCD monitor positioned 21 cm from the contralateral eye spanning 72° (in elevation) and

97° (in azimuth) of the animal's visual space. Stimuli consisted of sinusoidal gratings (spatial frequencies including 0.01, 0.025, and 0.04 cycles/° [Niell and Stryker, 2008]) drifting in 1 of up to 12 directions at a temporal frequency of 2 cycles/s. In cells where we compared two or more spatial frequencies, we observed no effect on the PSP integral, evoked firing rate, or OSI. For each trial, gratings were presented in the following manner: stationary (1 s)-moving (2 s)-stationary (1 s).

Gratings were presented in sequences according to their maximal difference (+210°). Jitter in the onset of the stimulus caused by the refresh rate of the monitor was compensated for by implementing a small photodiode in front of the screen, which allowed for alignment of the onset of the stimulus.

Electrophysiological Data Analysis

Evoked Responses

All data are expressed as mean ± SEM unless otherwise stated. We excluded direction-nonspecific onset responses by analyzing the membrane voltage during the second half of the 2 s drift. This analysis time window is expected to include evoked feed-forward and feed-back signals (Ringach et al., 1997). However, when the entire stimulus duration is analyzed, there remains a significant difference between CC and CT tuning for both orientation and direction ($p < 0.05$).

The membrane potential responses for each direction were determined by averaging across stimulus repetitions (four to six trials). For analysis of the subthreshold membrane potential, APs were clipped on each side of the peak at the level where the membrane potential variance (Vm standard deviation) equaled the mean variance in the absence of spiking. Linear interpolation was then used to join the membrane voltage traces. For the analysis of the peak depolarization (PSP_{Peak}, in mV), the most depolarized membrane potential value was used for directions in which evoked spiking was recorded. The analysis of the integral of the responses ($PSP_{Integral}$, in mV.s) was performed of average traces for each direction. The output response to drifting gratings was calculated by detecting action potentials in each trial and averaging the spiking rate for each direction.

For a given cell, the tuning profile was analyzed by first calculating the vector average of the responses (for $PSP_{Integral}$, PSP_{Peak}, or Output) for the 12 directions. The direction closest to the value of the vector average was then defined as the preferred direction. The response to the orthogonal direction was calculated as the average of the two sets of responses recorded for the Pref + $\pi/2$ (Ortho1) and Pref − $\pi/2$ (Ortho2). The null direction response is defined as the response recorded at Pref + π (Null direction). The orientation and direction indexes (OSI and DSI, respectively) are defined as follows: OSI = (Pref − Ortho)/(Pref + Ortho) and DSI = (Pref − Null)/(Pref + Null).

For experiments where the evoked synaptic potentials were injected into cells, the last 1 s of the response to four directions was used (Pref, Ortho1, Null, Ortho2). These were selected from three randomly chosen CC and CT cells. The amplitude of the current waveform to be injected was first determined by injecting the same cell type "input" and adjusting the current amplitude such that at least one spike could be evoked. The same current amplitude was then used for injections of the other "input" waveform.

Intrinsic Parameters

Current-voltage relationships were obtained from each neuron by injecting step currents ranging from −400 to 0 pA (in +50 pA steps of 600 ms) and depolarizing currents from 0 pA to two times the rheobase (in +25 pA steps of 600 ms). Biophysical parameters of recorded neurons were analyzed using Igor Pro 5 (http://www.wavemetrics.com). Briefly, the initial instantaneous frequency of the first two action potentials (F1) was extracted from rheobase to two times the rheobase, and the slope of the relationship between the initial instantaneous frequency and the current injected was calculated (F1-I slope). In addition, the initial instantaneous frequency and the instantaneous frequency 200 ms after the onset of the current injection at two times the rheobase were calculated ($F1_{2xRb}$ and $F200_{2xRb}$, respectively) in order to assess the early accommodation index of spiking (E_{acc} index = $((F1_{2xRb} - F200_{2xRb})/F1_{2xRb}) \times 100$). For quantification of membrane potential sag, hyperpolarizing current steps (−400 pA, 600 ms) were injected. To determine the sag potential amplitude, the most negative membrane potential value determined in the first 100 ms (peak) was compared to the average membrane potential recorded during the last 200 ms (steady state) of the current step period. The absolute difference in these two values was used as the sag potential amplitude. When comparing these intrinsic properties for morphologically identified CC (n = 6) and CT cells (n = 10), statistically significant differences were observed for all four parameters ($p < 0.05$). On average, there was found to be no significant difference in input resistance between CC and CT cells.

To classify cells a cluster analysis (Cauli et al., 2000; Thorndike, 1953; Ward, 1963) using $F1_{2xRb}$, the F1-I slope, the E_{acc} index and sag potential amplitude was performed. Cells sharing similar parameters are expected to be close to one another in multidimensional Euclidean space. The number of clusters was defined using the Thorndike method (Thorndike, 1953), by comparing the within-cluster linkage distances.

All data are expressed as mean ± SEM unless otherwise indicated. Student's t test and Wilcoxon tests were used to determine the significance of normally and nonnormally distributed data, respectively.

Morphological Reconstructions and Analysis

For tissue processing-related information, see the Supplemental Information. For neuronal reconstructions, recorded neurons were either (1) filled with biocytin and mice immediately perfused or (2) loaded via the patch pipette with a DNA vector to drive expression of GFP. In the latter case, mice were returned to their home cage for up to 72 hr, after which they were anesthetized and transcardially perfused. For tracing of GFP-labeled cells, the cells were first immunostained as described in the Supplemental Information. Cells were reconstructed in 3D using Neurolucida (MBF Bioscience) under an Olympus BX61 at magnifications ranging from 4 to 100×. Final 3D reconstructions were analyzed in Neurolucida Explorer (MBF Bioscience). The field volume of dendrites and axons was calculated by computing the 3D convex hull, which is the convex volume enclosed between the neuronal process ends. To further characterize neuronal processes, we plotted the number of intersections between processes and concentric spheres of the gradually increased radius (+10 μm) centered at the cell body (Sholl, 1953).

Dendritic and Axonal Density Maps

Cells were first centered according to the location of the soma, then aligned with respect to the pial surface. Dendritic and axonal trees were then separately exported as vectorial models from Neurolucida to vrml or wavefront obj files. The files were opened in a 3D graphic software (v. 2.68, The Blender Foundation, http://www.blender.org), and model lines were converted to mesh lines and then to tubes of identical diameter. Finally, the meshes were converted to a 3D image stack (Grothausmann et al., 2012). Stacks were then opened in ImageJ (Fiji, Wayne Rasband, NIH) using the MetaImage reader/writer plugin, converted to 32 bits image stacks and low pass filtered. Stacks of each cell type were averaged and the result projected as an integral in the coronal plane. Finally, images were scaled by the ratio of the integral of the resultant stack and the average total length of processes in the given cell type. The movies of density map rotations were produced with a custom version of the ImageJ built-in 3D-projector plugin. The plugin was modified to work with 32 bit images and produce projections as sums.

Estimates of Spine Density

For spine counting, either biocytin-filled or GFP-expressing cells were imaged using wide-field (Olympus BX61, 100×/1.25 numerical aperture [NA]) or confocal (Leica SP5, 40×/1.3 NA) microscopy. For each cell, spines were manually tagged in between three and seven 80-μm-long dendritic segments. The length of each dendritic segment was extracted on the basis of either Neurolucida reconstructions or using a plugin (Simple Neurite Tracer) in Image J. For each cell, we sampled at least one dendritic segment per cortical layer.

Image Processing and Cell Counting

Fixed whole brains were embedded in 4% agar and placed under a two-photon microscope containing an integrated vibrating microtome and a motorized x-y-z stage (Osten and Margrie, 2013; Ragan et al., 2012). Coronal images were acquired via three optical pathways (red, green, and blue) as a set of 6 by 9 tiles with a resolution of 1(X) × 1(Y) μm obtained every 5 μm (Z) using an Olympus 10× objective (NA = 0.6) mounted on a piezoelectric element (Physik Instrumente).

Following acquisition, image tiles were stitched using Fiji and custom routines, including a custom version of the Fiji stitching plugin (Preibisch et al.,

2009) allowing the parallel processing of several image planes for higher throughput. Briefly, the illumination profile was computed from the average of all tiles across the brain and used to normalize the individual tiles that were then stitched together using a combination of the readout from the microscope stage and cross-correlations.

Cells were manually counted and their coordinates recorded in whole-brain image stacks. The coordinates of each marked cell were then used to position markers (red spheres) in the whole-brain image stack. The brain regions were determined using a standard mouse brain atlas (Franklin and Paxinos, 2008). For CC, CT, and cre-NTSR1 RV-labeling, we counted 138 ± 21, 338 ± 116, and 4,088 ± 945 cells, respectively. In 2 of the 11 data sets, fixed whole brains were immediately sliced using a standard vibratome, antibody-stained, and then imaged using a confocal microscope (1.8(X) × 1.8(Y) × 5(Z) μm, 10× and 20× objective; Leica SP5). Cell counting in these cases were manually performed on individual coronal slices.

For combined single-cell physiology and rabies virus tracing experiments from CC neurons, labeled presynaptic cells were identified and counted for nine different brain regions located in the ipsilateral hemisphere and include V1, thalamus (different nuclei pooled), hippocampal formation, cortical associational areas (including temporal associational cortex and parietal cortex) secondary visual cortex (including lateral and medial), retrosplenial cortex, and auditory cortex (primary and secondary). In addition we occasionally found cells in the white matter. For histograms, all of these regions (excluding V1, thalamus, V2, and RSP) are represented by the "others" category. In the contralateral hemisphere, cells were only found in the secondary visual cortex.

For CT experiments, presynaptic cells were found in additional areas and include the hypothalamus, somatosensory, motor, cingulate cortices, and contralateral V1. For histograms, all of these additional regions were included (excluding V1, thalamus, V2, and RSP) and are represented by the "others" category.

For cre-NTSR1 tracing, cells were found in all of the above CT-related brain areas. In addition, some cells were found in over 30 other regions (not described). All of these regions (excluding thalamus, V2, and RSP) were allocated to the "other" category for cre-NTSR1 connectivity analysis.

SUPPLEMENTAL INFORMATION

Supplemental Information includes Supplemental Experimental Procedures, three figures, and three movies and can be found with this article online at http://dx.doi.org/10.1016/j.neuron.2014.08.001.

ACKNOWLEDGMENTS

The authors thank Elliot Gerard, Miles Wells, and Jessica Newberry Le Vay for performing neuronal reconstructions; Danielle Carmignac for performing immunohistochemistry; and Bruno Pichler for MATLAB routines. We are grateful to Sepideh Keshavarzi, Sonja Hofer and Andreas Schaefer for critical comments on the manuscript. We thank Sebastian Seung in whose laboratory the ETN construct and the TagRFP-T rabies virus was produced by I.R.W.; E.A.R. was supported by a Sir Henry Wellcome Fellowship. This work was funded by an Investigator Award from the Wellcome Trust (T.W.M.). T.W.M. is supported by the MRC MC_U1175975156.

Accepted: July 29, 2014
Published: August 28, 2014

REFERENCES

Allman, J., Miezin, F., and McGuinness, E. (1985). Stimulus specific responses from beyond the classical receptive field: neurophysiological mechanisms for local-global comparisons in visual neurons. Annu. Rev. Neurosci. *8*, 407–430.

Angelo, K., Rancz, E.A., Pimentel, D., Hundahl, C., Hannibal, J., Fleischmann, A., Pichler, B., and Margrie, T.W. (2012). A biophysical signature of network affiliation and sensory processing in mitral cells. Nature *488*, 375–378.

Angelucci, A., Levitt, J.B., Walton, E.J., Hupe, J.M., Bullier, J., and Lund, J.S. (2002). Circuits for local and global signal integration in primary visual cortex. J. Neurosci. *22*, 8633–8646.

Berezovskii, V.K., Nassi, J.J., and Born, R.T. (2011). Segregation of feedforward and feedback projections in mouse visual cortex. J. Comp. Neurol. *519*, 3672–3683.

Binzegger, T., Douglas, R.J., and Martin, K.A. (2004). A quantitative map of the circuit of cat primary visual cortex. J. Neurosci. *24*, 8441–8453.

Bock, D.D., Lee, W.C., Kerlin, A.M., Andermann, M.L., Hood, G., Wetzel, A.W., Yurgenson, S., Soucy, E.R., Kim, H.S., and Reid, R.C. (2011). Network anatomy and in vivo physiology of visual cortical neurons. Nature *471*, 177–182.

Bolz, J., and Gilbert, C.D. (1986). Generation of end-inhibition in the visual cortex via interlaminar connections. Nature *320*, 362–365.

Bortone, D.S., Olsen, S.R., and Scanziani, M. (2014). Translaminar inhibitory cells recruited by layer 6 corticothalamic neurons suppress visual cortex. Neuron *82*, 474–485.

Briggman, K.L., Helmstaedter, M., and Denk, W. (2011). Wiring specificity in the direction-selectivity circuit of the retina. Nature *471*, 183–188.

Briggs, F. (2010). Organizing principles of cortical layer 6. Front. Neural Circuits *4*, 3.

Brumberg, J.C., Hamzei-Sichani, F., and Yuste, R. (2003). Morphological and physiological characterization of layer VI corticofugal neurons of mouse primary visual cortex. J. Neurophysiol. *89*, 2854–2867.

Callaway, E.M. (1998). Local circuits in primary visual cortex of the macaque monkey. Annu. Rev. Neurosci. *21*, 47–74.

Carey, R.G., and Neal, T.L. (1985). The rat claustrum: afferent and efferent connections with visual cortex. Brain Res. *329*, 185–193.

Cauli, B., Porter, J.T., Tsuzuki, K., Lambolez, B., Rossier, J., Quenet, B., and Audinat, E. (2000). Classification of fusiform neocortical interneurons based on unsupervised clustering. Proc. Natl. Acad. Sci. USA *97*, 6144–6149.

Chadderton, P., Schaefer, A.T., Williams, S.R., and Margrie, T.W. (2014). Sensory-evoked synaptic integration in cerebellar and cerebral cortical neurons. Nat. Rev. Neurosci. *15*, 71–83.

Chen, T.W., Wardill, T.J., Sun, Y., Pulver, S.R., Renninger, S.L., Baohan, A., Schreiter, E.R., Kerr, R.A., Orger, M.B., Jayaraman, V., et al. (2013). Ultrasensitive fluorescent proteins for imaging neuronal activity. Nature *499*, 295–300.

Clark, B.J., Bassett, J.P., Wang, S.S., and Taube, J.S. (2010). Impaired head direction cell representation in the anterodorsal thalamus after lesions of the retrosplenial cortex. J. Neurosci. *30*, 5289–5302.

De Pasquale, R., and Sherman, S.M. (2011). Synaptic properties of corticocortical connections between the primary and secondary visual cortical areas in the mouse. J. Neurosci. *31*, 16494–16506.

De Pasquale, R., and Sherman, S.M. (2013). A modulatory effect of the feedback from higher visual areas to V1 in the mouse. J. Neurophysiol. *109*, 2618–2631.

Denk, W., Briggman, K.L., and Helmstaedter, M. (2012). Structural neurobiology: missing link to a mechanistic understanding of neural computation. Nat. Rev. Neurosci. *13*, 351–358.

Douglas, R.J., and Martin, K.A. (2007). Mapping the matrix: the ways of neocortex. Neuron *56*, 226–238.

Feldmeyer, D., Brecht, M., Helmchen, F., Petersen, C.C., Poulet, J.F., Staiger, J.F., Luhmann, H.J., and Schwarz, C. (2013). Barrel cortex function. Prog. Neurobiol. *103*, 3–27.

Fino, E., and Yuste, R. (2011). Dense inhibitory connectivity in neocortex. Neuron *69*, 1188–1203.

Franklin, K.B.J., and Paxinos, G. (2008). The Mouse Brain. In Stereotaxic Coordinates, Compact, Third Edition (New York: Elsevier).

Glickfeld, L.L., Andermann, M.L., Bonin, V., and Reid, R.C. (2013). Corticocortical projections in mouse visual cortex are functionally target specific. Nat. Neurosci. *16*, 219–226.

Gong, S., Doughty, M., Harbaugh, C.R., Cummins, A., Hatten, M.E., Heintz, N., and Gerfen, C.R. (2007). Targeting Cre recombinase to specific neuron

populations with bacterial artificial chromosome constructs. J. Neurosci. *27*, 9817–9823.

Grothausmann, R., Fiechter, S., Beare, R., Lehmann, G., Kropf, H., Vinod Kumar, G.S., Manke, I., and Banhart, J. (2012). Automated quantitative 3D analysis of faceting of particles in tomographic datasets. Ultramicroscopy *122*, 65–75.

Helmstaedter, M., Briggman, K.L., Turaga, S.C., Jain, V., Seung, H.S., and Denk, W. (2013). Connectomic reconstruction of the inner plexiform layer in the mouse retina. Nature *500*, 168–174.

Hirsch, J.A., Gallagher, C.A., Alonso, J.M., and Martinez, L.M. (1998). Ascending projections of simple and complex cells in layer 6 of the cat striate cortex. J. Neurosci. *18*, 8086–8094.

Hofer, S.B., Ko, H., Pichler, B., Vogelstein, J., Ros, H., Zeng, H., Lein, E., Lesica, N.A., and Mrsic-Flogel, T.D. (2011). Differential connectivity and response dynamics of excitatory and inhibitory neurons in visual cortex. Nat. Neurosci. *14*, 1045–1052.

Huang, T.N., Chuang, H.C., Chou, W.H., Chen, C.Y., Wang, H.F., Chou, S.J., and Hsueh, Y.P. (2014). Tbr1 haploinsufficiency impairs amygdalar axonal projections and results in cognitive abnormality. Nat. Neurosci. *17*, 240–247.

Hubel, D.H., and Wiesel, T.N. (1968). Receptive fields and functional architecture of monkey striate cortex. J. Physiol. *195*, 215–243.

Hupé, J.M., James, A.C., Payne, B.R., Lomber, S.G., Girard, P., and Bullier, J. (1998). Cortical feedback improves discrimination between figure and background by V1, V2 and V3 neurons. Nature *394*, 784–787.

Iurilli, G., Ghezzi, D., Olcese, U., Lassi, G., Nazzaro, C., Tonini, R., Tucci, V., Benfenati, F., and Medini, P. (2012). Sound-driven synaptic inhibition in primary visual cortex. Neuron *73*, 814–828.

Katz, L.C. (1987). Local circuitry of identified projection neurons in cat visual cortex brain slices. J. Neurosci. *7*, 1223–1249.

Kätzel, D., Zemelman, B.V., Buetfering, C., Wölfel, M., and Miesenböck, G. (2011). The columnar and laminar organization of inhibitory connections to neocortical excitatory cells. Nat. Neurosci. *14*, 100–107.

Kerlin, A.M., Andermann, M.L., Berezovskii, V.K., and Reid, R.C. (2010). Broadly tuned response properties of diverse inhibitory neuron subtypes in mouse visual cortex. Neuron *67*, 858–871.

Ko, H., Hofer, S.B., Pichler, B., Buchanan, K.A., Sjöström, P.J., and Mrsic-Flogel, T.D. (2011). Functional specificity of local synaptic connections in neocortical networks. Nature *473*, 87–91.

Kozloski, J., Hamzei-Sichani, F., and Yuste, R. (2001). Stereotyped position of local synaptic targets in neocortex. Science *293*, 868–872.

Kumar, P., and Ohana, O. (2008). Inter- and intralaminar subcircuits of excitatory and inhibitory neurons in layer 6a of the rat barrel cortex. J. Neurophysiol. *100*, 1909–1922.

Ledergerber, D., and Larkum, M.E. (2010). Properties of layer 6 pyramidal neuron apical dendrites. J. Neurosci. *30*, 13031–13044.

Mao, T., Kusefoglu, D., Hooks, B.M., Huber, D., Petreanu, L., and Svoboda, K. (2011). Long-range neuronal circuits underlying the interaction between sensory and motor cortex. Neuron *72*, 111–123.

Margrie, T.W., Brecht, M., and Sakmann, B. (2002). In vivo, low-resistance, whole-cell recordings from neurons in the anaesthetized and awake mammalian brain. Pflugers Arch. *444*, 491–498.

Marshel, J.H., Mori, T., Nielsen, K.J., and Callaway, E.M. (2010). Targeting single neuronal networks for gene expression and cell labeling in vivo. Neuron *67*, 562–574.

Martin, K.A., and Schröder, S. (2013). Functional heterogeneity in neighboring neurons of cat primary visual cortex in response to both artificial and natural stimuli. J. Neurosci. *33*, 7325–7344.

Maruyama, Y., and Ito, H. (2013). Diversity, heterogeneity and orientation-dependent variation of spike count correlation in the cat visual cortex. Eur. J. Neurosci. *38*, 3611–3627.

Marx, M., and Feldmeyer, D. (2013). Morphology and physiology of excitatory neurons in layer 6b of the somatosensory rat barrel cortex. Cereb. Cortex *23*, 2803–2817.

Mease, R.A., Krieger, P., and Groh, A. (2014). Cortical control of adaptation and sensory relay mode in the thalamus. Proc. Natl. Acad. Sci. USA *111*, 6798–6803.

Mercer, A., West, D.C., Morris, O.T., Kirchhecker, S., Kerkhoff, J.E., and Thomson, A.M. (2005). Excitatory connections made by presynaptic cortico-cortical pyramidal cells in layer 6 of the neocortex. Cereb. Cortex *15*, 1485–1496.

Nauhaus, I., Benucci, A., Carandini, M., and Ringach, D.L. (2008). Neuronal selectivity and local map structure in visual cortex. Neuron *57*, 673–679.

Niell, C.M., and Stryker, M.P. (2008). Highly selective receptive fields in mouse visual cortex. J. Neurosci. *28*, 7520–7536.

Oberlaender, M., de Kock, C.P., Bruno, R.M., Ramirez, A., Meyer, H.S., Dercksen, V.J., Helmstaedter, M., and Sakmann, B. (2012). Cell type-specific three-dimensional structure of thalamocortical circuits in a column of rat vibrissal cortex. Cereb. Cortex *22*, 2375–2391.

Oh, S.W., Harris, J.A., Ng, L., Winslow, B., Cain, N., Mihalas, S., Wang, Q., Lau, C., Kuan, L., Henry, A.M., et al. (2014). A mesoscale connectome of the mouse brain. Nature *508*, 207–214.

Olsen, S.R., Bortone, D.S., Adesnik, H., and Scanziani, M. (2012). Gain control by layer six in cortical circuits of vision. Nature *483*, 47–52.

Osten, P., and Margrie, T.W. (2013). Mapping brain circuitry with a light microscope. Nat. Methods *10*, 515–523.

Petersen, C.C., and Sakmann, B. (2000). The excitatory neuronal network of rat layer 4 barrel cortex. J. Neurosci. *20*, 7579–7586.

Pichon, F., Nikonenko, I., Kraftsik, R., and Welker, E. (2012). Intracortical connectivity of layer VI pyramidal neurons in the somatosensory cortex of normal and barrelless mice. Eur. J. Neurosci. *35*, 855–869.

Preibisch, S., Saalfeld, S., and Tomancak, P. (2009). Globally optimal stitching of tiled 3D microscopic image acquisitions. Bioinformatics *25*, 1463–1465.

Priebe, N.J., Lisberger, S.G., and Movshon, J.A. (2006). Tuning for spatiotemporal frequency and speed in directionally selective neurons of macaque striate cortex. J. Neurosci. *26*, 2941–2950.

Ragan, T., Kadiri, L.R., Venkataraju, K.U., Bahlmann, K., Sutin, J., Taranda, J., Arganda-Carreras, I., Kim, Y., Seung, H.S., and Osten, P. (2012). Serial two-photon tomography for automated ex vivo mouse brain imaging. Nat. Methods *9*, 255–258.

Rancz, E.A., Franks, K.M., Schwarz, M.K., Pichler, B., Schaefer, A.T., and Margrie, T.W. (2011). Transfection via whole-cell recording in vivo: bridging single-cell physiology, genetics and connectomics. Nat. Neurosci. *14*, 527–532.

Reid, R.C. (2012). From functional architecture to functional connectomics. Neuron *75*, 209–217.

Reis Marques, T., Taylor, H., Chaddock, C., Dell'acqua, F., Handley, R., Reinders, A.A., Mondelli, V., Bonaccorso, S., Diforti, M., Simmons, A., et al. (2014). White matter integrity as a predictor of response to treatment in first episode psychosis. Brain *137*, 172–182.

Ringach, D.L., Hawken, M.J., and Shapley, R. (1997). Dynamics of orientation tuning in macaque primary visual cortex. Nature *387*, 281–284.

Roth, M.M., Helmchen, F., and Kampa, B.M. (2012). Distinct functional properties of primary and posteromedial visual area of mouse neocortex. J. Neurosci. *32*, 9716–9726.

Salin, P.A., and Bullier, J. (1995). Corticocortical connections in the visual system: structure and function. Physiol. Rev. *75*, 107–154.

Schwarz, C., and Bolz, J. (1991). Functional specificity of a long-range horizontal connection in cat visual cortex: a cross-correlation study. J. Neurosci. *11*, 2995–3007.

Sholl, D.A. (1953). Dendritic organization in the neurons of the visual and motor cortices of the cat. J. Anat. *87*, 387–406.

Sohya, K., Kameyama, K., Yanagawa, Y., Obata, K., and Tsumoto, T. (2007). GABAergic neurons are less selective to stimulus orientation than excitatory

neurons in layer II/III of visual cortex, as revealed by in vivo functional Ca2+ imaging in transgenic mice. J. Neurosci. *27*, 2145–2149.

Thomson, A.M. (2010). Neocortical layer 6, a review. Front Neuroanat. *4*, 13.

Thomson, A.M., and Bannister, A.P. (2003). Interlaminar connections in the neocortex. Cereb. Cortex *13*, 5–14.

Thorndike, R.L. (1953). Who belongs in the family? Psychometrika *18*, 267–276.

Usrey, W.M., and Fitzpatrick, D. (1996). Specificity in the axonal connections of layer VI neurons in tree shrew striate cortex: evidence for distinct granular and supragranular systems. J. Neurosci. *16*, 1203–1218.

Wang, Q., and Burkhalter, A. (2007). Area map of mouse visual cortex. J. Comp. Neurol. *502*, 339–357.

Ward, J.H. (1963). Hierarchical grouping to optimize an objective function. J. Am. Stat. Assoc. *58*, 236–244.

Wickersham, I.R., Finke, S., Conzelmann, K.K., and Callaway, E.M. (2007). Retrograde neuronal tracing with a deletion-mutant rabies virus. Nat. Methods *4*, 47–49.

Xu, W., Huang, X., Takagaki, K., and Wu, J.Y. (2007). Compression and reflection of visually evoked cortical waves. Neuron *55*, 119–129.

Yoshimura, Y., Dantzker, J.L., and Callaway, E.M. (2005). Excitatory cortical neurons form fine-scale functional networks. Nature *433*, 868–873.

Zarrinpar, A., and Callaway, E.M. (2006). Local connections to specific types of layer 6 neurons in the rat visual cortex. J. Neurophysiol. *95*, 1751–1761.

Zhang, Z.W., and Deschênes, M. (1997). Intracortical axonal projections of lamina VI cells of the primary somatosensory cortex in the rat: a single-cell labeling study. J. Neurosci. *17*, 6365–6379.

Zhang, R., Wei, Q., Kang, Z., Zalesky, A., Li, M., Xu, Y., Li, L., Wang, J., Zheng, L., Wang, B., et al. (2014). Disrupted brain anatomical connectivity in medication-naïve patients with first-episode schizophrenia. Brain Struct. Funct. Published online January 22, 2014. http://dx.doi.org/10.1007/s00429-014-0706-z.

Note Added in Proof

Since the acceptance of this manuscript, a paper has been published demonstrating that activation of layer 6 CT cells leads to the direct activation of layer 5a neurons: Kim, J., Matney, C.J., Blankenship, A., Hestrin, S., Brown, S.P. (2014). Layer 6 corticothalamic neurons activate a cortical output layer, layer 5a. J. Neurosci. 34, 9656–9664.

Neuron, Volume *83*

Supplemental Information

The Stimulus Selectivity and Connectivity of Layer Six Principal Cells Reveals Cortical Microcircuits Underlying Visual Processing

Mateo Vélez-Fort, Charly V. Rousseau, Christian J. Niedworok, Ian R. Wickersham, Ede A. Rancz, Alexander P.Y. Brown, Molly Strom, and Troy W. Margrie

Supplemental Figures, Movies, Experimental Procedures and References

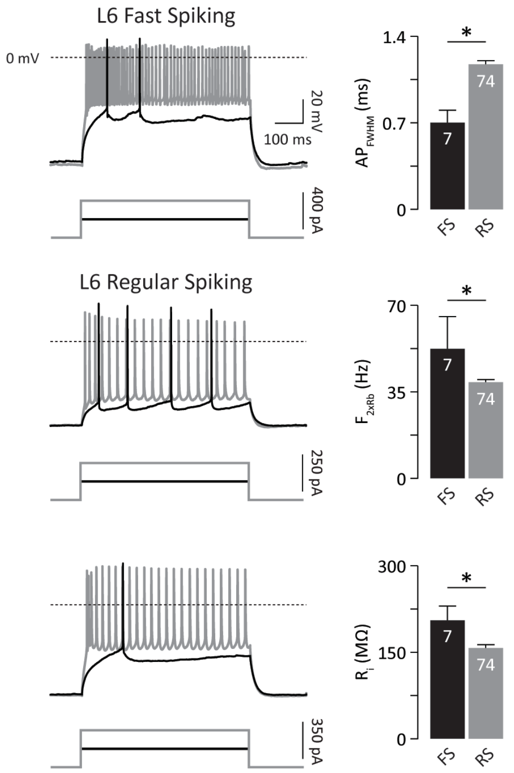

Supplementary Figure 1

Figure S1 (related to Figure 1): Intrinsic properties of recorded fast and regular spiking L6 cells Left column: Example traces recorded from fast (top) and regular spiking (middle and bottom) neurons recorded at the rheobase (black) and 2x the rheobase (grey). Right: Bar graphs comparing the AP width at half the peak amplitude (AP_{FWHM}), average frequency of firing at 2x the rheobase (F_{2xRb}) and input resistance (R_i).

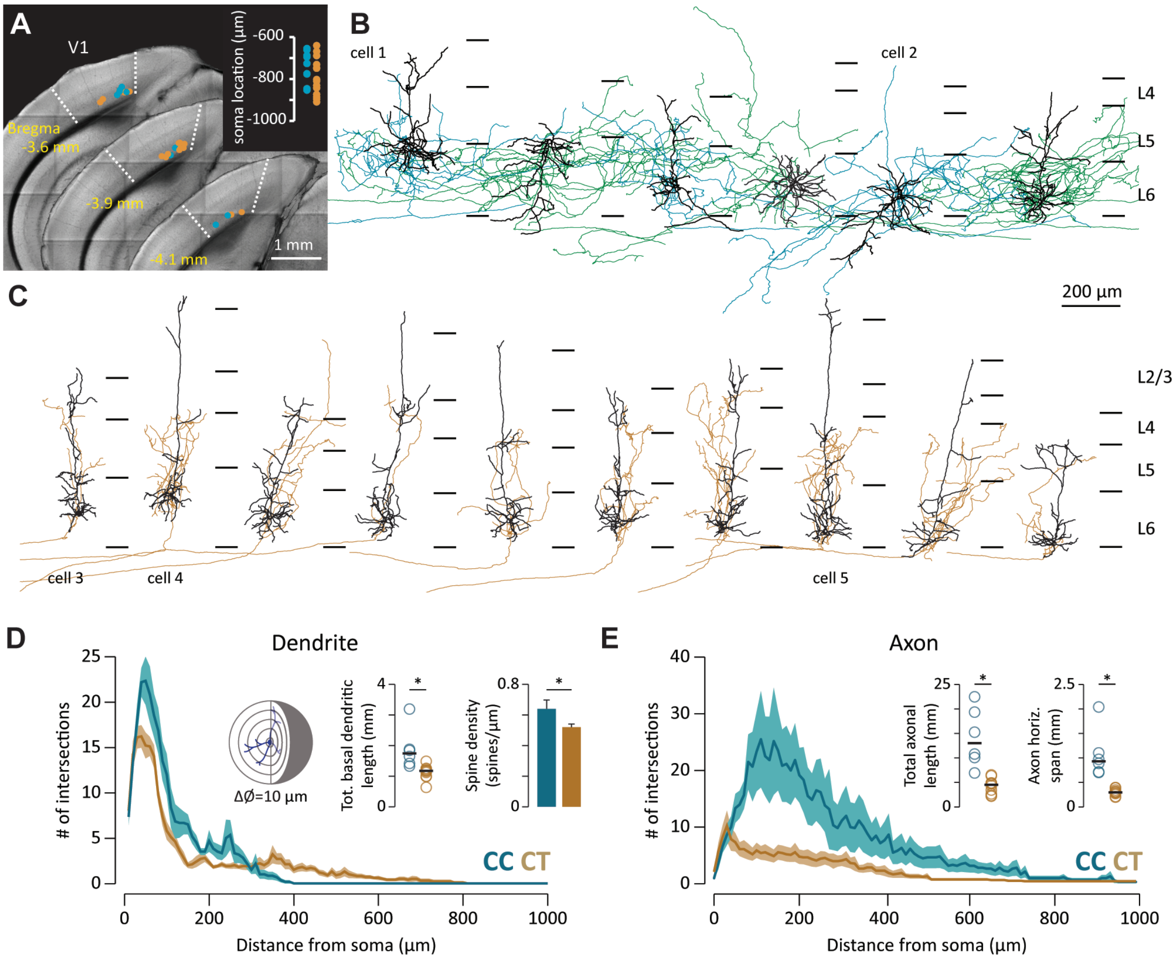

Supplementary Figure 2

Figure S2 (related to Figure 1): Morphological classification of *in vivo*-recorded L6 principal cells

(**A**) Three consecutive "reference brain" slices showing the location of the reconstructed cells in B and C recorded from individual brains. Inset indicates the shortest distance of the cell soma from the pia. (**B**) Reconstructions of recorded neurons with dendrites that do not project beyond layer 4 and have elaborate axonal branching within the cortex. (**C**) Morphologies of recorded cells that extended their dendrites beyond layer 4 and always projected their axons into the thalamic tract. (**D**) Sholl analysis showing the number of concentric sphere crossings made by CC (blue) and CT (brown) dendritic arborizations plotted against their distance from the cell soma. Insets show the total basal dendritic for each reconstructed CC and CT cell (left), the horizontal bar indicates the median value. Right, histogram of the mean spine density for CC (n = 3, 12 segments) and CT cells (n = 4, 20 segments). (**E**) Sholl analysis showing the number of concentric sphere crossings made by CC (blue) and CT (brown) axonal arborizations plotted against their distance from the cell soma. Insets show the total axonal length (left) and medial-lateral (horizontal) span for CC versus CT axons. The median is indicated by the horizontal bar.

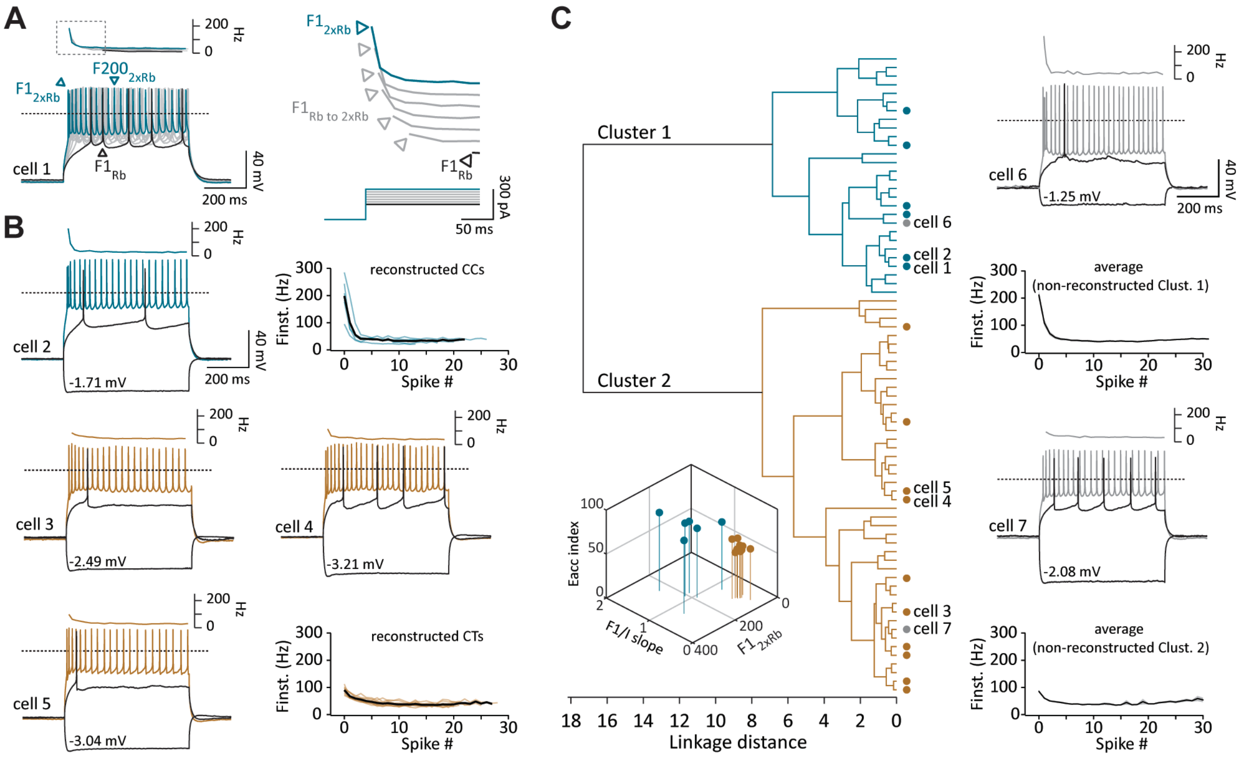

Supplementary Figure 3

Figure S3 (related to Figure 1): Biophysical classification of recorded cells verified by morphological identity

(**A**) Example of membrane voltage traces from a reconstructed CC cell showing the biophysical parameters used to perform the cluster analysis in (C). $F1_{Rb}$ = instantaneous frequency of the first two spikes evoked by current step injection at the rheobase. $F1_{2xRb}$ = instantaneous frequency of the first two spikes evoked at 2x the rheobase. $F200_{2xRb}$ = the instantaneous firing frequency 200 ms after the onset of the current injection at 2x the rheobase. Top: Plot of the instantaneous firing frequency evoked by current steps ranging from the rheobase (black) to 2x the rheobase (blue). Right: Example traces highlighted by the dashed box showing the relationship between F1 and the current step amplitude. (**B**) Example membrane voltage traces from one CC (blue) and three CT (brown) reconstructed cells showing spiking at the rheobase and 2x the rheobase. Above: Plot of the instantaneous firing frequency evoked by current step injection at 2x the rheobase. Insets: The average instantaneous firing rates recorded at 2x the rheobase plotted against spike number for all reconstructed CC (blue) and CT cells (brown). (**C**) Left: Result of the cluster analysis using the membrane sag and the three firing parameters indicated in the 3D plot (insert). Clustered cells with known morphology are indicated by filled blue (CC) and brown (CT) circles. Right: The average instantaneous firing rates recorded at 2x the rheobase plotted against spike number for all cells in the respective cluster (except the reconstructed cells). Example traces from individual non-reconstructed cells selected from cluster 1 (top) and cluster 2 (below) showing spiking at rheobase and 2x the rheobase.

Movie S1 (related to Figure 1): 3D projection showing the dendritic density for CC and CT cells. Movies are the sum of dendritic reconstructions of six and ten in vivo-recorded cells (CC and CT respectively) centered on the neuronal somata and vertically aligned according to the shortest distance to the pia surface. The dendritic density is expressed in false color (see Figure 1B).

Movie S2 (related to Figure 1): 3D projection showing the axonal density for CC and CT cells. Movies are the sum of the axonal reconstructions of six and ten in vivo-recorded cells (CC and CT respectively) centered on the neuronal somata and vertically aligned according to the shortest distance to the pia surface. The axonal density is expressed in false color (see Figure 1B).

Movie S3 (related to Figures 5 and 6): Raw images of retrograde labeled RFP-expressing cells. Top: Stacks of 50 coronal images (every 5 µm (Z)) showing modified rabies-infected neurons within the deep layers of V1 after a single-CC cell rabies tracing experiment (left: native RFP, right: spheres localized using the coordinates of manually counted RFP-expressing cells). Bottom: Stacks showing cells within the deep layers of V1 after a single-CT cell rabies tracing experiment (left: native RFP, right: spheres localized using the coordinates of manually counted RFP-expressing cells).

Experimental Procedures

Tissue processing

Deeply anaesthetised mice were transcardially perfused with cold PB (0.1 M) followed by 4% paraformaldehyde (PFA) in PB (0.1 M) and the brain left overnight in 4% PFA at 4 °C. Coronal brain sections (50 - 150 µm) were either cut directly on a vibrating microtome (Leica VT1200S or Microm HM 650V) or obtained following serial tomography, then rinsed in PBS. Fluorescence images were acquired on a Leica SP1, SP5 or a Zeiss 510 laser-scanning confocal microscope. For experiments containing biocytin in the intracellular solution, slices were either treated for streptavidin or 3,3-diaminobenzidine (DAB) labeling. For streptavidin labeling, slices were first treated with 5% bovine serum albumin and 2% Triton-X100 in PBS for 12 hours then incubated in a PBS solution containing 0.5% Alexa-488-conjugated streptavidin (Sigma-Aldrich), 5% bovine serum albumin and 0.1% Triton-X for 12 hours at 4˚C. For the DAB procedure, slices were incubated in H_2O_2 (3% wt/vol) for 30 minutes then rinsed with PB and incubated in a solution containing 0.1% triton-X100 and avidin-biotinylated horseradish peroxidase (Vector ABC staining kit, Vector Lab) for 12 hours at 4˚C. Finally, slices were rinsed with PB and incubated for 30 minutes in DAB solution; H_2O_2 (3% wt/vol) was added under visual control until neuronal cell processes were visible then slices were rinsed with PB and mounted (see below). For immunostaining against GFP and RFP, chicken anti-GFP (Life Technologies 1:500) and rabbit anti-tRFP primary antibodies (Evrogen 1:1000; 12 hours at RT) were visualized with goat DyLight-488 anti-chicken (Abcam 1:500) and goat Alexa Fluor-555 anti-rabbit (Life Technologies 1:500, 12 hours at RT) secondary antibodies, respectively. After thorough washing in PB, slices were mounted with an anti-fading medium (Mowiol 4-88, Calbiochem + 2.5% DABCO 33-LV, Sigma-Aldrich).

Assignment of cortical layers

Cortical layers in V1 were manually assigned using either DAPI staining or the background fluorescence (cell nuclei appear as shadows) to qualitatively use cell densities to identify laminar boundaries. Boundary assignment was independently performed by four postgraduate and postdoctoral cortical neuroscientists in the laboratory. The maximum discrepancy in layer assignment was 52 µm which was at the layer 5/6 boundary. Across all layer boundaries the average maximum discrepancy was 26 µm.

Plasmids and viruses

Production

Plasmids pSil-ETN-IRES2-EGFP (protein expressed from ETN is a fusion of the extracellular and trans-membrane domains of the rabies glycoprotein from the CVS-N2c strain and the intracellular domain from the SADB19 glycoprotein, Addgene 58831) and pSil-TVA800-IRES2-EGFP were made by replacing GFP in pCA-b-GFPm5 silencer 3 (generous gift of Hendrik Wildner and François Guillemot) by the indicated cassettes to enable expression in neurons from CAGG promoter. Human XIAP cDNA was made by RT-PCR (Quantitect, Qiagen) amplification using oligonucleotides (5' ccacgtctctggccaagatgacttttaacagttttg and 5' ccacgtctcggatccgctaatggaattcaatcctg) and cloned into pCA-b-GFPm5 silencer 3 replacing GFP (Addgene, 58832). The plasmid pAAV-EF1a-Flex-GT is from Addgene (26198). The plasmid pAAV-EF1a-Flex-C-RG was made by replacing GT in pAAV-EF1a-Flex-GT with Cerulean-E2A-SADB19 rabies glycoprotein (RV-G) (Addgene, 49101). pAAV2/8 was obtained from Penn Vector Core and pHelper and AAV293 cells were purchased from Agilent Technologies. AAV virus was prepared from transfection of 5 x 100 mm dishes as per manufacturer's protocol (Agilent Technologies) and were purified from cell pellets as described (Guo et al., 2012) through to PEG precipitation. Virus containing pellets were re-suspended by trituration and overnight incubation at 4^oC in 1xHBSS (Invitrogen), extracted with an equal volume of chloroform, and concentrated using

Amicon Ultra-Ultracel 100 K filters (Millipore) to a final volume of approximately 100 µl. EnvA pseudotyped SADB19 rabies virus expressing tagRFP in place of the RG was prepared from EnvA-RV-tagRFP by infecting cells expressing TVA950 and RG (expression plasmids were generous gifts from Melvyn Yap and Martin Schwarz, respectively) to amplify the unpseudotyped virus. This stock was used to re-pseudotype virus with EnvA according to published methods (Wickersham et al., 2007). All reagents for cell culture were from Invitrogen, BHK cells and BHK-EnvARGCD cells were a generous gift of Martin Schwartz.

Injections

For virus injection, long-shanked, volume-calibrated pipettes (Blaubrand) were pulled and tip-filled using negative pressure (Cetin et al., 2006). To ensure the injection site was close to the recorded cell, pipettes were mounted onto the recording manipulator on the same setup used for whole-cell recording and plasmid delivery. Approximately 50 nl of modified rabies virus solution was injected under 30-50 mbar of positive pressure for 3 minutes. The pipette was withdrawn, Kwik-Sil re-applied and the animal sutured and recovered. For cre-NTSR1 mice, a first injection of approximately 20 nl of the helper virus (see below) was followed 2 days later by a second injection of approximately 50 nl of the modified rabies virus solution. Post-RV injection mice were maintained for 10-12 days.

Supplemental References

Cetin, A., Komai, S., Eliava, M., Seeburg, P.H., and Osten, P. (2006). Stereotaxic gene delivery in the rodent brain. Nat. Protoc. *1*, 3166-3173.
Guo, P., El-Gohary, Y., Prasadan, K., Shiota, C., Xiao, X., Wiersch, J., Paredes, J., Tulachan, S., and Gittes, G.K. (2012). Rapid and simplified purification of recombinant adeno-associated virus. J. Virol. Methods *183*, 139-146.
Wickersham, I.R., Finke, S., Conzelmann, K.K., and Callaway, E.M. (2007). Retrograde neuronal tracing with a deletion-mutant rabies virus. Nat. Methods *4*, 47-49.
